# Immunoinformatic Analysis of T- and B-Cell Epitopes for SARS-CoV-2 Vaccine Design

**DOI:** 10.3390/vaccines8030355

**Published:** 2020-07-03

**Authors:** Dongliang Wang, Jinhui Mai, Wenfeng Zhou, Wanting Yu, Yang Zhan, Naidong Wang, Neal D. Epstein, Yi Yang

**Affiliations:** 1 Hunan Provincial Key Laboratory of Protein Engineering in Animal Vaccines, Laboratory of Functional Proteomics (LFP), Research Center of Reverse Vaccinology (RCRV), College of Veterinary Medicine, Hunan Agricultural University, Changsha 410128, China; dongliangwang@stu.hunau.edu.cn (D.W.); jinhuimai@stu.hunau.edu.cn (J.M.); wenfengzhou@stu.hunau.edu.cn (W.Z.); 1906390265@pku.edu.cn (W.Y.); yangzhan@hunau.edu.cn (Y.Z.); naidongwang@hunau.edu.cn (N.W.); 2 State Key Laboratory of Membrane Biology, Biodynamic Optical Imaging Center (BIOPIC), School of Life Sciences, Peking University, Beijing 100871, China; 3 Cell and Developmental Biology Center, NHLBI, NIH, Bethesda, MD 20892, USA

**Keywords:** SARS-CoV-2, S protein, B-cell and T-cell epitopes, vaccine

## Abstract

Currently, there is limited knowledge about the immunological profiles of Severe Acute Respiratory Syndrome Coronavirus 2 (SARS-CoV-2). We used computer-based immunoinformatic analysis and the newly resolved 3-dimensional (3D) structures of the SARS-CoV-2 S trimeric protein, together with analyses of the immunogenic profiles of SARS-CoV, to anticipate potential B-cell and T-cell epitopes of the SARS-CoV-2 S protein for vaccine design, particularly for peptide-driven vaccine design and serological diagnosis. Nine conserved linear B-cell epitopes and multiple discontinuous B-cell epitopes composed of 69 residues on the surface of the SARS-CoV-2 trimeric S protein were predicted to be highly antigenic. We found that the SARS-CoV-2 S protein has a different antigenic profile than that of the SARS-CoV S protein due to the variations in their primary and 3D structures. Importantly, SARS-CoV-2 may exploit an immune evasion mechanism through two point mutations in the critical and conserved linear neutralization epitope (overlap with fusion peptide) around a sparsely glycosylated area. These mutations lead to a significant decrease in the antigenicity of this epitope in the SARS-CoV-2 S protein. In addition, 62 T-cell epitopes in the SARS-CoV-2 S protein were predicted in our study. The structure-based immunoinformatic analysis for the SARS-CoV-2 S protein in this study may improve vaccine design, diagnosis, and immunotherapy against the pandemic of COVID-19.

## 1. Introduction

The outbreak of the coronavirus disease 2019 (COVID-19) is caused by a novel coronavirus named Severe Acute Respiratory Syndrome Coronavirus 2 (SARS-CoV-2) [1]. By 16 June 2020, SARS-CoV-2 has been reported in 216 nations and has resulted in 7,941,791 confirmed cases (https://www.who.int/emergencies/diseases/novel-coronavirus-2019).

**Figure 1:**
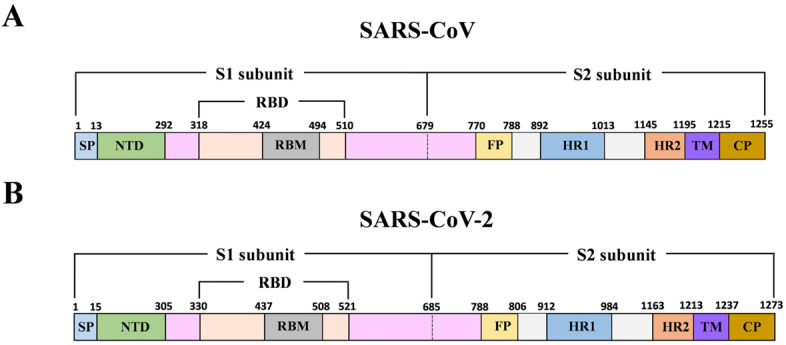
Schematic of Severe Acute Respiratory Syndrome Coronavirus (SARS-CoV) and SARS-CoV-2 S protein. (**A**, **B**) Schematic of SARS-CoV and SARS-CoV-2 genome encoding spike protein. SP: signal peptide; NTD: N-terminal domain; RBD: receptor-binding domain; RBM: receptor-binding motif; FP: fusion peptide; HR1 and HR2: heptad repeat regions 1 and 2; TM: transmembrane; CP: cytoplasmic tail region.

Coronavirus (CoV) belongs to the family of Coronaviridae, and it is an enveloped, positive-sense single-stranded RNA virus. Both SARS-CoV-2 and SARS-CoV fit into the subgenus of *Sarbecovirus* within the genus of *Betacoronavirus* (Beta-CoV), based on phylogenetic tree analysis [1,2,3]. The viral genome, approximately 30 kb in size, encodes four structural proteins including the spike (S), envelope (E), membrane (M), and nucleocapsid (N) proteins. The S protein is composed of an ectodomain, a transmembrane domain (TM), and a short cytoplasmic tail region (CP) (Figure 1). The S protein forms homotrimers on the viral membrane surface. The ectodomain of S protein is composed of two subunits (S1 and S2) which are responsible for host cell receptor engagement and membrane fusion, respectively. The S1 subunit contains an NH2-terminal domain (NTD) and the carboxyl-terminal domain (CTD), which is also called the receptor-binding domain (RBD). Coronaviruses may use both domains to interact with a variety of host cell receptors for cell entry. For instance, both SARS-CoV and SARS-CoV-2 use the RBD to specifically bind angiotensin-converting enzyme 2 (ACE2) on host cells for virus entry [4,5,6]. Molecular modeling has suggested that the SARS-CoV-2 receptor-binding domain (RBD) has a stronger binding affinity to ACE2 [7]. Middle East Respiratory Syndrome Coronavirus (MERS-CoV) uses its RBD to bind to host cell receptor dipeptidyl peptidase 4 (DPP4) [8]. Some other members, such as porcine epidemic diarrhea virus (PEDV), employ the NTD to interact with a sugar moiety on the host cell membrane for binding and entry [9]. SARS-CoV receptor (ACE2) binding to RBD of the S1 subunit induces subsequent conformational changes of the S2 subunit, which leads to the fusion of the cell and the virus membranes [10,11]. The S2 subunit contains two heptad repeat domains (HR1 and HR2) that play a critical role in SARS-CoV membrane fusion with target cells. 

SARS-CoV infection triggers a series of humoral and cellular immune responses, including the production of high titers of specific neutralizing antibodies and specific cytotoxic T lymphocyte responses to SARS-CoV [12,13]. The S protein is the major structural antigenic component through which effective protective immunity is raised against virus infection. A vaccine based on the S protein could elicit antibodies to neutralize virus infection by blocking virus fusion and entry. The SARS-CoV-2 S protein shares a high degree of similarity to the SARS-CoV S protein [14,15], and it also binds in similar fashion to the human ACE2 receptor and thus is likely to employ a similar cell entry mechanism [4,16]. As such, the S protein is an effective antigenic component for SARS-CoV-2 vaccine design and development. However, currently, there is little or limited information about the immunogenic profiles of SARS-CoV-2 and the immune responses against SARS-CoV-2. Despite this, computer-based immunoinformatics [17], together with the recent progress on the 3-dimensional (3D) SARS-CoV-2 S protein [14,15,18,19,20,21], offers a powerful strategy providing rational and rapid guidelines for the design and development of effective vaccines against this emerging infectious disease.

In this study, the close genetic relationship of SARS-CoV-2 with other members of the genus of Beta-CoV, especially with SARS-CoV, prompted us to explore the potential immunogenic profiles of SARS-CoV-2 for vaccine design and development. We used computer-based immunoinformatic analysis, together with analyses of the immunogenic profiles of SARS-CoV, to anticipate potential B-cell and T-cell epitopes of the S protein of SARS-CoV-2 for vaccine design, particularly peptide-driven vaccine design, immunotherapy, and serological diagnosis.

## 2. Materials and Methods 

### 2.1. B-Cell Epitope Prediction

Linear B-cell epitopes of the SARS-CoV-2 S protein were predicted by BepiPred 2.0 in IEDB (BepiPred 2.0., Immune Epitope Database and Analysis Resource, National Institute of Allergy and Infectious Diseases, Bethesda, MD, USA) with a threshold of 0.55 (corresponding specificity > 0.817 and sensitivity < 0.292), and only the epitopes with more than 8 residues were considered for subsequent antigenicity analysis. Antigenicity was evaluated via the VaxiJen v2.0 server online tool (VaxiJen v2.0., The Jenner Institute, Oxford, UK) [22]. Discontinuous B-cell epitopes were predicted via the DiscoTope 2.0 server tool in IEDB with a default threshold of −3.7 (corresponding specificity > 0.75 and sensitivity < 0.47), based on the 3-dimensional (3D) structures of the SARS-CoV-2 S protein (PDB ID: 6VYB, B chain) and the SARS-CoV-2 S protein RBD (PDB ID: 6M0J, B chain).

### 2.2. T-Cell Epitope Prediction

CD8 T-cell epitopes were predicted based on the Net MHC pan 4.0 algorithm in IEDB with a peptide size of 9 residues, and the 8 most frequent HLA class I alleles (HLA-A*01:01, HLA-A*02:01, HLA-A*03:01, HLA-A*11:01, HLA-A*24:02, HLA-B*07:02, HLA-B*08:01, and HLA-B*40:01) in the worldwide population (phenotypic frequency > 10%) were selected [23]. The top 1% of peptides with high scores were chosen for subsequent immunogenicity evaluation, which was analyzed by the VaxiJen v2.0 server. For CD4 T-cell epitope prediction, an IEDB-recommended 2.22 algorithm based on 7 alleles (DRB1*0301, DRB1*0701, DRB1*0501, DRB3*0101, DRB3*0202, DRB4*0101, and DRB5*0101) [24] at a default 15-aa peptide was used with a median consensus percentile of prediction threshold ≤ 20, as recommended. 

### 2.3. SARS-CoV S Protein Epitope Acquisition

B-cell and T-cell epitopes of the SARS-CoV S protein were searched in IEDB by using IEDB’s Immunobrowser tool. To identify B- and T-cell epitopes tested by experiments, only the epitopes with the response frequency (RF) values more than 0.5 were considered as positive. 

### 2.4. Peptide Modeling and Molecular Docking

3D structures of all peptides were modelled via the PEP-FOLD3 online server [25]. All the peptides were docked to the MHC I molecules HLA-B7 (PDB ID: 3VCL) and HLA-A*01:01 (PDB ID: 4NQV) via the PatchDock rigid-body docking server based on the defined threshold [26]. The docking transformation with good molecular shape complementarity was selected based on the geometry docking algorithm in PatchDock, and then scoring and refining of the docked complexes were performed using the FireDock server [27,28]. The docking complexes with high global energy, attractive van der Waals (vdW) energy, and hydrogen-bonding energy were used for subsequent analysis. Protein–peptide connection was examined via LigPlot+ v.2.2, and Pymol (Version 1.8.4.0, Schrödinger, Inc, New York, NJ, USA) was used to analyze docked complexes. 

## 3. Results

### 3.1. B-Cell Epitope Prediction and Analysis of Spike Glycoprotein

**Figure 2:**
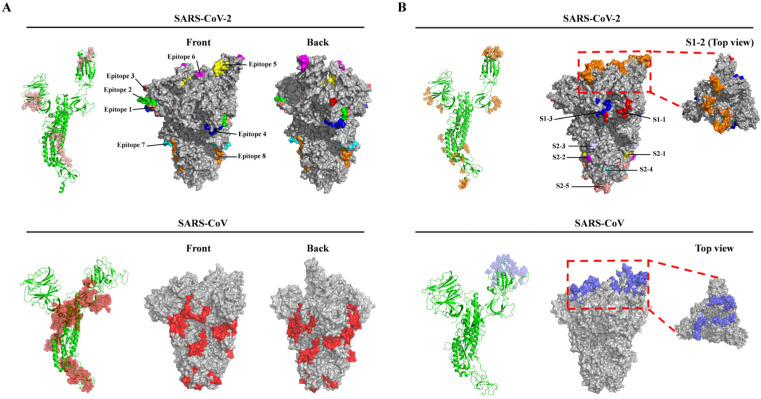
Localization of linear (**A**) and discontinuous (**B**) B-cell epitopes of the SARS-CoV-2 and SARS-CoV S proteins. (**A**) The predicted linear B-cell epitopes of SARS-CoV-2 (top) and the known linear B-cell epitopes of SARS-CoV (bottom) in the monomeric (left, ribbon model) and on the trimeric (middle and right, surface model) S proteins. Eight predicted epitopes of the SARS-CoV-2 S protein are shown as salmon in the monomeric S protein or distinct colors on the trimeric S protein surface (1: salmon; 2: green; 3: red; 4: blue; 5: yellow; 6: magenta; 7: cyan; 8: orange). The epitopes of the SARS-CoV S protein are shown as red in the monomeric and trimeric S protein surfaces. (**B**) The predicted discontinuous B-cell epitopes of SARS-CoV-2 (top) and the known discontinuous B-cell epitopes (Epitope ID: 77442, 77444, and 910052) of SARS-CoV (bottom) in the monomeric (left, ribbon model) and on the trimeric (middle and right, surface model) S proteins. The epitopes of SARS-CoV-2 are shown as orange in monomer and distinct colors on the trimeric S protein surface (S1–1: red; S1–2: orange; S1–3: blue; S2–1: yellow; S2–2: magenta; S2–3: light blue; S2–4: cyan; S2–5: salmon). The epitopes of the SARS-CoV S protein are shown as blue in the monomeric S protein and on the trimeric S protein surface. The boxes with red dashed lines indicate the membrane-distal termini of both trimeric S proteins and the top views are on the right. Note: Three-dimensional (3D) structure models of the SARS-CoV-2 S protein (PDB ID: 6VYB) and SARS-CoV S protein (PDB ID: 6ACD) were retrieved from the PDB database.

In all, 17 potential linear B-cell epitopes were predicted by the BepiPred 2.0 program (Table S1, Supplementary Materials), and nine linear B-cell epitopes were chosen for further analysis after their antigenicity was evaluated via the VaxiJen v2.0 program, based on the scores (Table 1). All the predicted B-cell epitopes were localized to a strictly conserved region and shared 100% identity throughout the 138 SARS-CoV-2 isolates. Structure simulations demonstrated that all the nine epitopes were located on the surfaces of either the monomer or the trimer of the S protein (Figure 2A, top panel). Of note, of the nine epitopes, epitope 5 (^405^DEVRQIAPGQTGKI^418^) was localized to the RBD and epitope 6 (^441^LDSKVGGN^448^) to the RBM of the SARS-CoV-2 S protein (Table 1). We also reviewed seven dominant linear B-cell epitopes of the SARS-CoV S proteins based on previous experimental tests and response frequency (see Methods). Of the seven epitopes, two epitopes were identical throughout all the 87 SARS-CoV isolates and four were highly conserved (≥93.1% SARS-CoV isolates had identical epitopes) (Table S2, Supplementary Materials). These results suggested that the majority of linear B-cell epitopes of the S protein were highly conserved in SARS-CoV and SARS-CoV-2 isolates, respectively (Table 1 and Table S2). It is worth noting that one epitope (^786^QILPDPLKPTKRSFIEDLLFNKVTLA^811^) located in the S2 subunit of the SARS-CoV S protein is an important linear B-cell epitope capable of eliciting the production of a neutralizing antibody (NAb) identified in patients who recovered from SARS-CoV infection (Table S2) [13]. In addition, we also predicted linear B-cell epitopes of the SARS-CoV S protein, and six of the seven dominant linear B-cell epitopes were predicted by BepiPred 2.0, since the six dominant B-cell epitopes had overlapping sequences with their counterparts in the predicted epitope pool, thereby supporting BepiPred 2.0 as a reliable and powerful tool for predicting linear B-cell epitopes. Finally, the comparison of the epitope sequences revealed that there were no overlapping sequences between the nine potential linear B-cell epitopes of SARS-CoV-2 and the seven dominant linear B-cell epitopes of SARS-CoV (Table 1 and Table S2), suggesting that the immunogenetic profile of the SARS-CoV-2 S protein may be different from that of SARS-CoV.

Besides the linear B-cell epitopes, 69 residues on the surface of the S protein of the SARS-CoV-2 were predicted to form the multiple discontinuous B-cell epitopes (Table 2). Furthermore, based on the primary structure and 3D structure of the trimeric S protein, these residues were mainly distributed within eight regions (Table 2 and Figure 2B). Notably, region S1–2 containing 35 residues accounted for more than half of the residues (35/69) comprising the discontinuous B-cell epitopes, and these 35 residues of region S1–2 were all located in the RBD. Furthermore, 31 of the 35 residues were in the RBM (region S1–2 in Table 2). This result suggested that the RBD, particularly the RBM, was highly antigenic. In addition, among the discontinuous epitope(s) of region S1–2, 10 residues (G417, G446, Y449, Q493, G496, Q498, T500, N501, G502, and Y505) were identified as the key residues contributing to the binding to the host receptor ACE2 [19] (Table 2). In region S2–2, two residues (P793 and I794) were located in the fusion peptide (FP) and exposed on the surface of the S2 subunit (Table 2 and Figure 2B, top panel). Therefore, antibodies targeting these two regions may block the virus binding to the host cell receptor and the subsequent membrane fusion between virus and host cell. In addition, sequence alignments revealed that these 69 residues were strictly conserved among the S proteins of the 138 SARS-CoV-2 isolates, except that a point mutation (P1143L, region S2–5, Table 2) occurred in the Australia/QLD02/2020 strain. Although this mutation did not change the secondary structure (Figure S1A, Supplementary Materials), it caused a slight increase in the antigenicity (the antigenic scores increasing from 0.558 to 0.565). Indeed, as shown in Figure S1B, the longer side chain of ^1143^L caused an apparent alteration of the surface structure.

Next, we examined all the discontinuous B-cell epitopes of the SARS-CoV S protein deposited in the IEDB database, and three main epitopes (Epitope ID: 77442, 77444, and 910052) were obtained from the database (Table S3, Supplementary Materials). Furthermore, these conformational epitopes could be recognized by a variety of neutralizing mAbs (80R, m396 and S230) in previous studies [29,30]. 3D structure analyses revealed that the residues among the three discontinuous B-cell epitopes were exclusively mapped onto the RBD surface of the S1 subunit, suggesting that the RBD of the SARS-CoV S protein is also highly antigenic. We also compared the common residues comprising the discontinuous epitopes within the RBDs of both the RBDs of SARS-CoV and SARS-CoV-2 S proteins. Only seven residues (Y449, N450, L492, G496, T500, G502, and Y505 of the SARS-CoV-2 S protein) were present in the identical positions of both S proteins (Table 2). Therefore, the three mAbs (80R, m396, and S230) recognizing the RBD of the SARS-CoV S protein hardly bound the SARS-CoV-2 RBD, although the RBDs of both SARS-CoV and SARS-CoV-2 exhibit a high degree of 3D structural homology [14]. Altogether, compared to the SARS-CoV S protein, the SARS-CoV-2 S protein may have a distinct antigenic profile, although both viruses are closely related by phylogenetic analysis (Figures S2 and S3, Supplementary Materials).

### 3.2. T-Cell Epitope Prediction for the SARS-CoV-2 S Protein

In all, 40 peptides were predicted as the potential CD8 T-cell epitopes following analysis of peptide-MHC-I binding of the SARS-CoV-2 S protein using the Net MHC pan 4.0 server and their subsequent evaluation of antigenicity using VaxiJen v2.0 (Table S4, Supplementary Materials). Similarly, 22 potential CD4 T-cell epitopes were predicted to be present in the SARS-CoV-2 S protein (Table 3). Three of the 62 predicted T-cell epitopes (CD8 and CD4 T-cell epitopes above) of the SARS-CoV-2 S protein have been reported as T-cell epitopes of the SARS-CoV S protein in previous studies [31,32,33,34] (Table S5, Supplementary Materials). The first and second were CD8 T-cell epitopes (^493^PYRVVVLSF^501^, Epitope ID: 50166; ^1174^NLNESLIDL^1182^, Epitope ID: 44814), while the first one was located in the RBD of the SARS-CoV S protein, which is known to be important for receptor binding and virus entry [35]. The third one was encompassed in one of the CD4 T-cell epitopes (^993^QLIRAAEIRASANLAATK^1010^, Epitope ID: 100428) of the SARS-CoV S protein (the epitope highlighted with underline showed the predicted CD4 T-cell epitope derived from the SARS-CoV-2 S protein). 

### 3.3. Molecular Docking of Predicted CD8 T-Cell Epitopes with HLA Alleles 

**Figure 3:**
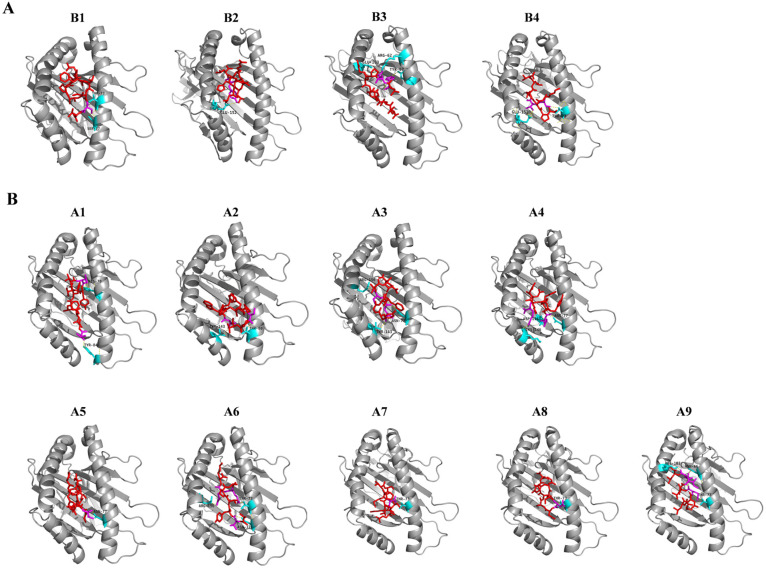
Molecular docking analysis of SARS-CoV-2 peptides (shown as red) to human HLA-B7 (**A**) and HLA-A*01:01 (**B**) protein (shown as gray). The interacting residues of peptides are shown as magenta and the HLA-B7 and HLA-A*01:01 interacting residues are shown as cyan. B1 to B4 and A1 to A9 correspond to the peptide with information provided in Table 4.

Before molecular docking with HLA molecules, the 3D structures of the 40 potential CD8 T-cell epitopes were modelled via PEP-FOLD3. Only the best 3D model of each epitope was chosen for the subsequent molecular docking with HLA molecules. Among the 40 epitopes, four were docked into HLA-B7 and nine were docked into HLA-A*01:01. For the four peptide-HLA-B7 molecular docking, the binding efficiency of each epitope was evaluated by the global and vdW energies, which were computed ranging from −11.55 to −26.14 kcal/mol and −18.15 to −25.38 kcal/mol, respectively (Table 4). All the four peptides were predicted to be well docked into the groove of the HLA-B7 molecule and formed stable hydrogen bonds with the residues in the groove of the HLA (Figure 3A). Notably, both T73 and E152 in the HLA-B7 groove frequently interacted with the epitopes via hydrogen bonding within 3.1Å (Figure S4A, Supplementary Materials). Furthermore, the global and vdW energies of the nine peptide-HLA-A*01:01 dockings ranged from −12.90 to −46.66 kcal/mol and −12.10 to −25.71 kcal/mol, respectively (Table 4). Of these nine peptides, five peptides showed high binding affinities and the other four peptides showed even higher binding affinities with HLA-A*01:01. Hydrogen bonds less than 3.1Å were frequently observed in docking complexes. T73, N77, T143, and R156 within the groove were the major residues interacting with these peptides and formed stable complexes (Figure 3B and Figure S4B).

## 4. Discussion

Vaccination is the most effective medical strategy against a variety of infectious diseases. Unfortunately, to date, no vaccine against coronavirus-associated diseases has been approved by the FDA for use in humans. Therefore, a vaccine against COVID-19 is urgently needed to control the pandemic caused by the highly contagious SARS-CoV-2. The lack of knowledge about SARS-CoV-2 immunogenic profiles and immune responses is a challenge to vaccine design and development. The S protein is a leading potential target for vaccine design for either SARS-CoV or SARS-CoV-2 infection because of its strong immunogenicity and its roles in virus attachment and cell entry [16,36]. Importantly, the S protein of SARS-CoV is capable of inducing the production of neutralizing antibodies (NAbs), which have been found in convalescent plasma samples from SARS patients [13] and in animal models [37,38]. Therefore, antibodies targeting the S protein, particularly the RBD/RBM or the S2 fusion machinery, may exhibit neutralizing activity against SARS-CoV-2 infections. SARS-CoV-2 and SARS-CoV belong to the same genus of the *Betacoronavirus* and both of them, together with the three coronaviruses from bat, show a very close genetic relationship in the evolution of the virus (Figure S2, Supplementary Materials). However, the similarity of the immunogenic properties of these viruses remains to be determined. Several immunological questions are critical: “Are the immunogenic profiles of SARS-CoV-2 and SARS-CoV as similar as the genetic relationship shown in the phylogenetic trees?”; “Do the immunogenic properties of both viruses differ significantly from each other?”; and “Can the NAbs raised against SARS-CoV provide effective protection against the infection of SARS-CoV-2?”. We sought to identify the potential B-cell and T-cell epitopes of the SARS-CoV-2 S protein by using various state-of-the-art tools. The results improve our understanding of S protein immunogenesis and vaccine design. 

Nine linear B-cell epitopes were predicted and localized to the surface of the SARS-CoV-2 S protein (Table 1), while seven linear B-cell epitopes of the SARS-CoV S protein have been confirmed by previous investigations [13,39,40,41,42]. The two groups of epitopes do not share any similarities, even though the S proteins from both viruses are close to each other in their primary structure. One critical linear B-cell epitope (^786^QILPDPLKPTKRSFIEDLLFNKVTLA^811^) of the SARS-CoV S protein was reported to be recognized by NAbs obtained from convalescent SARS patients in a previous report [13]. Another group also reported that an epitope (^803^LLFNKVTLADAGFMKQYGECLGDINA^828^) was able to induce the production of NAbs in animal models [41]. Both epitopes localize to the S2 subunit and have a nine-residue overlap from position 803 to 811. Furthermore, two of our predicted linear B-cell epitopes (predicted via BepiPred 2.0 in IEDB) were also found to map to the region between 786Q–828A (data not shown), suggesting that the region (786Q–828A) is an epitope-rich region of the SARS-CoV S protein. The function of this region in the S2 subunit is still unknown, but we note that the region has a three-residue overlap with the fusion peptide (FP) from 770 to 788 (Figure 1), and a proteolytic cleavage site (S2′) upstream of the fusion peptide is conserved in all known coronaviruses [43]. Therefore, antibodies targeting this epitope-rich site could potentially block FP function in membrane fusion during virus cell entry. Comparison of the SARS-CoV and SARS-CoV-2 S proteins reveals that the S2 subunit is structurally conserved and shares higher aa identity (~90%), than does the S1 subunit (~68%). Likewise, we also identified a homologous peptide (^804^QILPDPSKPSKRSFIEDLLFNKVTLA^829^) in the S2 subunit of the SARS-CoV-2 S protein, which differs by only two residues from the corresponding region in SARS-CoV (see the residues indicated by the underlines). However, this homologous peptide in the SARS-CoV-2 S protein does not qualify as a predicted linear B-cell epitope. We noticed that the two residues in SARS-CoV (792L and 795T) are replaced by residues with less bulky side chains in SARS-CoV-2 (810S and 813S), which may decrease the antigenicity of the peptides and support SARS-CoV-2 countering host immune surveillance and clearance. Indeed, we evaluated the antigenicity of both peptides using VaxiJen v2.0, and found that the antigenicity score of the linear B-cell epitope in the SARS-CoV S protein was 0.4121, almost double the score of the peptide in the SARS-CoV-2 S protein (0.2114). Although the antigenicity of this peptide in SARS-CoV-2 remains to be experimentally determined, and currently, most vaccine designs are focusing on the RBD of the S1 subunit, we speculate that a vaccine based on this epitope-rich region (786Q–828A) in the S2 subunit of the SARS-CoV S protein may also elicit broad NAbs that can cross-react with other virus members among this coronavirus family to provide broader protections (i.e., against simultaneous infections of both SARS-CoV and SARS-CoV-2). Very recently, Poh et al. used sera from COVID-19 convalescent patients to identify peptides eliciting NAbs from a pool constructed from overlapping sequences of the SARS-CoV-2 S protein [44]. One of the identified peptides (^809^PSKPSKRSFIEDLLFNKV^826^) is also from the S2 subunit, located near the fusion peptide of the SARS-CoV-2 S protein. However, as discussed above, the antigenicity of this peptide and its effect across human populations of different ages, genetic backgrounds, and immune status requires further evaluation before being used for vaccine design, due to its lower antigenicity score compared to the concomitant SARS-CoV S protein. In addition, S proteins of coronaviruses are decorated with an extensive glycan shield, which blocks neutralizing antibody recognition and presents a challenge for vaccine development. Walls et al. recently characterized the S glycan shield of the SARS-CoV S protein [43]. According to their result, this epitope-rich region (786Q–828A) located in a glycan hole that is sparsely glycosylated provides access to host protease for further proteolysis and subsequent induction of membrane fusion. Taken together, this epitope-rich region is an ideal target for SARS-CoV-2 vaccine design.

Besides these critical linear B-cell epitopes, multiple discontinuous conformational B-cell epitopes distributed throughout eight regions were located on the surface of the trimeric S protein (Table 2 and Figure 2B). Region S1–2 is one of the critical sites that could be targeted by NAbs since it is located in the RBD, and specifically on the surface of the RBM. Ten of the 35 residues among the conformational epitope(s)/regions are potentially involved in binding of the SARS-CoV-2 RBD to hACE2: (G417, G446, Y449, Q493, G496, Q498, T500, N501, G502, and Y505) (Table 2) [19]. It is possible that antibodies compete with hACE2 to bind the SARS-CoV-2 RBD, and thereafter block the interaction of the virus with the receptor and the subsequent virus cell entry. In addition, seven of the 35 residues (Y449, N450, L492, G496, T500, G502, and Y505) of the SARS-CoV-2 RBD are also identified in the SARS-CoV RBD. These are the key residues forming a conformational epitope recognized by NAbs in previous studies [45,46].

Cell-mediated immunity plays crucial roles in the response to virus infection as well as cancer therapy. CD8 cytotoxic T cells kill cells via T-cell receptor (TCR) recognition of the cognate peptide presented by MHC class I. Two critical CD8 T-cell epitopes (Table 4, A5 and A9 in Figure 3), previously reported in SARS-CoV studies, were also predicted as CD8 T-cell epitopes of the SARS-CoV-2 S protein in our study. Importantly, both the epitopes can be docked onto the HLA-A*01:01 allele in an energetically favorable manner (Table 4). These results strongly suggest that both of the CD8 T-cell epitopes are authentic epitopes of the SARS-CoV-2 S protein and are possibly involved in cell-mediated immune responses against SARS-CoV-2 infection. Currently, most vaccine designs of the virus focus on the NAb production elicited by the S protein, but T cell-mediated immunity (both CD8+ and CD4+ helper cells) against the viral infection deserves more attention. Furthermore, “human-like” T-cell epitopes in SARS-CoV-2 should be removed from the vaccine since these epitopes are able to activate Treg cells and suppress the immune response [47].

Epitope prediction via immunoinformatics has accelerated the identification of antigens capable of eliciting a strong immune protective response against pathogen infections. Likewise, it can remove deleterious epitopes from the antigen pool, which may cause antibody-dependent enhancement (ADE), cytokine storm, autoimmune responses, and pathological lesions. The authenticity and effectiveness of these predicted epitopes may be improved through the use of threshold scoring and further confirmed by in vitro experiments and animal models. Since epitope mapping of a new pathogen is time-consuming and laborious work, epitope prediction by immunoinformatics improves the efficiency of vaccine design and development. For instance, Gutiérrez et al. predicted cross-conserved T-cell epitopes of seven representative strains of Influenza A virus (IAV) in US swine herds [48]. Following the prediction, researchers in Tanja Opriessnig’s group designed and tested a DNA vaccine containing these predicted cross-conserved T-cell epitopes followed by an inactivated vaccine for boost. The new designed vaccine (prime-boosting regimen) exhibits an additive increase in cell-mediated immunity and an excellent clinical protection [49]. A pool of epitopes may be chosen as the core immunogen to develop various peptide-driven vaccines, such as a peptide vaccine, a DNA/RNA vaccine encoding these tandem epitopes, or a subunit vaccine via grafting these epitopes onto a defined nanoparticle, i.e., virus-like particles (VLPs). In contrast to the whole pathogen-based vaccine, the injurious side effects (i.e., ADE, cytokine storm, autoimmune responses, and pathological lesions) of these isolated epitopes can be evaluated in vitro or in animal models. Thereafter, the peptide-driven vaccine with the optimized epitope combination will be safer and more effective as a result of its more precise design guided by a variety of versatile immunoinformatic tools.

Cytokine storm is one of the most dangerous and potentially lethal sequelae of COVID-19 infection but the details of its onset and why it affects one patient rather than another remain unknown. Several possible pathways may be responsible for SARS-CoV-2-associated cytokine storm, but it is likely that a failure to initially suppress viral replication leads to severe tissue damage from an overwhelming infection and a subsequent uncontrolled immune response. The initial cytokine wave following SARS-CoV-2 infection comes from the innate immune response. Pattern recognition receptors (PRRs), such as toll-like receptors (TLR-3, -7, and -8), RIG-I-like receptors (RLRs), and NOD-like receptors (NLRs) recognize the viral RNA genome or its intermediates during replication. This recognition causes substantial releases of proinflammatory cytokines, such as TNF-α, IL-1β, and IL-6. Second, the proliferation of SARS-CoV-2 in host cells leads to a large amount of cell death, and the content of the dead cells release damage signals, which further amplify cytokine release leading to cytokine storm [50]. Lastly, cytokine storm may be exacerbated through a complement cascade after infection. The large number and combinatorial diversity of N-linked glycans on the surface of the SARS-CoV-2 S protein can be recognized by mannose-binding lectin (MBL), which is able to initiate the complement cascade and activate macrophages. Subsequently, these activated macrophages also release a large amount of cytokine, i.e., IL-1, IL-6, and TNF-α. Thus, a peptide-driven vaccine in the absence of viral genome and various glycan-conjugated antigens, theoretically, promises to limit inappropriate cytokine release. An autoimmune response induced by foreign antigens presents another safety issue for vaccine design. Generally, pathogens may evade host immunosurveillance through producing “host-like” B-cell or T-cell epitopes and activating self-reactive T-regulatory cells that suppress the immune response or induce tolerance to the pathogens. However, in the context of a vaccine formula (combinations of antigens and adjuvants), these “host-like” epitopes may reversibly activate to induce an autoimmune response. Recently, EpiVax, Inc., has launched a new immunoinformatic tool called JanusMatrix that helps to identify “human-like” epitopes from pathogens [47]. Removing these “human-like” epitopes from a vaccine formula further enhances the safety and efficacy of such vaccines. Compared to the use of the whole virus proteome, the epitopes predicted in our study are more easily evaluated by these newly developed tools such as JanusMatrix, which removes the “human-like” epitopes from the vaccine. Finally, ADE is a critical safety concern for vaccine design and development. In general, ADE is mediated by non-neutralizing antibodies binding to virus and then promoting virus cell entry via Fcγ receptors (FcγRs). Actually, ADE was discovered in the course of vaccinations (i.e., RSV and Dengue virus) and is difficult to predict. ADE has been reported in animal experiments during vaccine trials of SARS-CoV and MERS-CoV [51,52]. However, ADE during vaccination for SARS-CoV or MERS-CoV, let alone SARS-CoV-2, remains to be determined in human. Epitope prediction and in vitro immunological assays can aid scientists in the identification and removal of potential ADE-promoting B-cell epitopes. The refined pool of candidate vaccine antigens can then be more exhaustively tested in animal models for evidence of ADE.

## 5. Conclusions

The results presented in this study highlight SARS-CoV-2 evolution and the structure-relevant immune profiles of both S proteins (SARS-CoV-2 and SARS-CoV). This perspective improves vaccine design and immunotherapy and works to minimize the side effects of vaccination for SARS-CoV-2.

## Figures and Tables

**Table 1:** Predicted linear B-cell epitopes on the surface of the SARS-CoV-2 S protein.

Epitope	Position	Sequence	Length	VaxiJen Score	Identity	Domain or Motif
1	15–31	CVNLTTRTQLPPAYTNS	17	1.2219	100%	NTD
2	62–75	VTWFHAIHVSGTNG	14	0.5786	100%	NTD
3	141–152	LGVYYHKNNKSW	13	0.8156	100%	NTD
4	208–220	TPINLVRDLPQGF	13	0.4768	100%	NTD
5	405–418	DEVRQIAPGQTGKI	14	0.9312	100%	RBD
6	441–448	LDSKVGGN	8	0.8773	100%	RBM
7	657–664	NNSYECDI	8	0.6539	100%	S1 (C-terminal)
8	696–709	TMSLGAENSVAYSN	14	0.6780	100%	S2 (N-terminal)
9	1154–1169	KYFKNHTSPDVDLGDI	16	0.7333	100%	S2 (C-terminal)

Note: Residues in the epitopes that are present in the crystal structure of the SARS-CoV-2 trimeric S protein are underlined; otherwise, they were absent in the crystal structure.

**Table 2:** Predicted discontinuous B-cell epitopes of the SARS-CoV-2 S protein.

Region	Sequences	Domain/Motif
S1–1	K97, S98, K187, P209, N211, E281, N282	NTD
S1–2	T415, K417, D420, Y421, N439, N440, S443, K444, V445, G446, G447, N448, * Y449, * N450, L452, R454, K458, S459, N460, K462, S477, P491, * L492, Q493, S494, * G496, F497, Q498, P499, * T500, N501, * G502, V503, G504, * Y505	RBD
S1–3	N556, K558, L560, P561, F562, Q563, L582	C-terminal
S2–1	N703, S704, V705	N-terminal
S2–2	P793, I794	FP
S2–3	P809, S810, K811	Between FP and HR1
S2–4	Y917, E918	HR1
S2–5	Q1071, N1074, T1100, L1141, Q1142, P1143, E1144, L1145, D1146, S1147	Between HR1 and HR2

Residues in the epitopes that are involved in binding of the SARS-CoV-2 RBD to hACE2 are underlined. * indicates the residues that are present at the identical positions of both SARS-CoV and SARS-CoV-2 S proteins.

**Table 3:** Predicted MHC class-II peptides of the SARS-CoV-2 S protein.

Start	End	Length	Peptide	Median Consensus Percentile	VaxiJen Score
5	19	15	LVLLPLVSSQCVNLT	20	1.2086
52	66	15	QDLFLPFFSNVTWFH	16	0.4159
57	71	15	PFFSNVTWFHAIHVS	16	0.7656
113	127	15	KTQSLLIVNNATNVV	13	0.6303
116	130	15	SLLIVNNATNVVIKV	14	0.4707
139	153	15	PFLGVYYHKNNKSWM	20	0.6641
199	213	15	GYFKIYSKHTPINLV	18	0.9278
209	223	15	PINLVRDLPQGFSAL	15	0.6086
230	244	15	PIGINITRFQTLLAL	13	0.8877
239	253	15	QTLLALHRSYLTPGD	16	0.6708
263	277	15	AAYYVGYLQPRTFLL	16	0.6073
309	323	15	EKGIYQTSNFRVQPT	18	0.9243
312	326	15	IYQTSNFRVQPTESI	11	0.7459
345	359	15	TRFASVYAWNRKRIS	16	0.4963
390	404	15	LCFTNVYADSFVIRG	17	0.5950
506	520	15	QPYRVVVLSFELLHA	14	0.9109
821	835	15	LLFNKVTLADAGFIK	19	0.6327
896	910	15	IPFAMQMAYRFNGIG	6.5	1.2828
1013	1027	15	IRAAEIRASANLAAT	14	0.6785
1016	1030	15	AEIRASANLAATKMS	13	0.8255
1060	1074	15	VVFLHVTYVPAQEKN	20	1.1720
1212	1226	15	WPWYIWLGFIAGLIA	19	0.7293

The underlined epitope is also identified as a T-cell epitope of the SARS-CoV S protein.

**Table 4:** Molecular docking results of HLA-B7 and HLA-A*01:01 with MHC I peptides.

Number	Start	End	Peptide	Global Energy (kcal/mol)	vdW Energy (kcal/mol)	H-Bonding Energy (kcal/mol)	Interacting Residues	VaxiJen Score	Alleles
B1	714	722	IPTNFTISV	−26.14	−22.96	−0.83	Thr73, Ser77	0.882	HLA-B7
B2	241	249	LLALHRSYL	−25.81	−25.38	−2.01	Glu152	0.5241	
B3	269	277	YLQPRTFLL	−17.28	−21.98	−0.41	Arg62, Tyr67, Glu163	0.4532	
B4	526	534	GPKKSTNLV	−11.55	−18.15	−1.99	Thr73, Glu152	0.6828	
A1	1060	1068	VVFLHVTYV	−46.66	−21.71	−1.89	His70, Tyr84	1.5122	HLA-A*01:01
A2	57	65	PFFSNVTWF	−37.65	−25.71	−1.94	Thr80, Thr143	0.6638	
A3	755	763	QYGSFCTQL	−26.61	−20.71	−0.82	Asn77, Thr143, Arg156	1.2906	
A4	142	150	GVYYHKNNK	−26.01	−22.48	−0.62	Asn77, Asp116, Lys146	0.8264	
A5	507	515	PYRVVVLSF	−17.96	−16.51	−1.87	Asn77	1.0281	
A6	1065	1073	VTYVPAQEK	−14.92	−24.24	−3.52	Thr73, Asn77, Arg156	0.8132	
A7	725	733	EILPVSMTK	−13.80	−12.10	−1.87	Thr73	1.6842	
A8	706	714	AYSNNSIAI	−13.29	−13.02	−1.81	Thr73	0.8274	
A9	1192	1200	NLNESLIDL	−12.90	−19.85	−4.32	Asn66, Thr73, Arg163	0.6827	

The underlined epitopes are also identified as T-cell epitopes of the SARS-CoV S protein.

## Supplementary Materials

The following are available online at https://www.mdpi.com/2076-393X/8/3/355/s1, Figure S1: The effect of P1143L mutation on the secondary structure and predicted 3D structure, Figure S2: Neighbor-joining tree analysis of SARS-CoV-2, Figure S3: Maximum-likelihood tree analysis of SARS-CoV-2, Figure S4: (A) 2D graphical representation of interaction analyses between human HLA-B7 protein and MHC-I binding peptides, (B) 2D graphical representation of interaction analyses between human HLA-A*01:01 protein and MHC-I binding peptides, Figure S5: (A) Sequence variability of the S protein between RaTG13 and SARS-CoV-2, (B) Sequence variability of the S protein among 138 SARS-CoV-2 clinical isolates, Table S1: 17 predicted linear B-cell epitopes of the SARS-CoV-2 S protein using BepiPred 2.0, Table S2: Linear B-cell epitopes of the SARS-CoV S protein, Table S3: Discontinuous B-cell epitopes of the SARS-CoV S protein, Table S4: Predicted MHC class-I epitopes in the SARS-CoV-2 S protein, Table S5: MHC class I and class II epitopes identified in the SARS-CoV S protein, Table S6: Detailed information of 138 SARS-CoV-2 sequences, Table S7: Detailed information of reference coronavirus strains in this study, Table S8: Percentage of nucleotide sequence and amino acid sequence identity of SARS-CoV-2 and reference CoV strains, Table S9: Sequence variability among structural proteins of SARS-CoV-2 clinical isolates.

## References

[1] Zhu N., Zhang D., Wang W., Li X., Yang B., Song J., Zhao X., Huang B., Shi W., Lu R. (2020). A Novel Coronavirus from Patients with Pneumonia in China, 2019. N. Engl. J. Med..

[2] Wu F., Zhao S., Yu B., Chen Y.-M., Wang W., Song Z.-G., Hu Y., Tao Z.-W., Tian J.-H., Pei Y.-Y. (2020). A new coronavirus associated with human respiratory disease in China. Nature.

[3] Zhou P., Yang X.-L., Wang X.-G., Hu B., Zhang L., Zhang W., Si H.-R., Zhu Y., Li B., Huang C.-L. (2020). A pneumonia outbreak associated with a new coronavirus of probable bat origin. Nature.

[4] Wan Y., Shang J., Graham R., Baric R.S., Li F. (2020). Receptor recognition by novel coronavirus from Wuhan: An analysis based on decade-long structural studies of SARS. J. Virol..

[5] Letko M., Marzi A., Munster V. (2020). Functional assessment of cell entry and receptor usage for SARS-CoV-2 and other lineage B betacoronaviruses. Nat. Microbiol..

[6] Li W., Moore M.J., Vasilieva N., Sui J., Wong S.K., Berne M.A., Somasundaran M., Sullivan J.L., Luzuriaga K., Greenough T.C. (2003). Angiotensin-converting enzyme 2 is a functional receptor for the SARS coronavirus. Nature.

[7] Chen Y., Guo Y., Pan Y., Zhao Z.J. (2020). Structure analysis of the receptor binding of 2019-nCoV. Biochem. Biophys. Res. Commun..

[8] Raj V.S., Mou H., Smits S.L., Dekkers D.H.W., Müller M.A., Dijkman R., Muth D., Demmers J.A.A., Zaki A., Fouchier R.A.M. (2013). Dipeptidyl peptidase 4 is a functional receptor for the emerging human coronavirus-EMC. Nature.

[9] Liu C., Tang J., Ma Y., Liang X., Yang Y., Peng G., Qi Q., Jiang S., Li J., Du L. (2015). Receptor usage and cell entry of porcine epidemic diarrhea coronavirus. J. Virol..

[10] Wong S.K., Li W., Moore M.J., Choe H., Farzan M. (2004). A 193-Amino Acid Fragment of the SARS Coronavirus S Protein Efficiently Binds Angiotensin-converting Enzyme 2. J. Biol. Chem..

[11] Li F., Berardi M., Li W., Farzan M., Dormitzer P.R., Harrison S.C. (2006). Conformational States of the Severe Acute Respiratory Syndrome Coronavirus Spike Protein Ectodomain. J. Virol..

[12] Xu X., Gao X.-M. (2004). Immunological Responses against SARS-Coronavirus Infection in Humans. Cell. Mol. Immunol..

[13] Zhong X., Yang H., Guo Z.-F., Sin W.-Y.F., Chen W., Xu J., Fu L., Wu J., Mak C.-K.G., Cheng C.-S.S. (2005). B-Cell Responses in Patients Who Have Recovered from Severe Acute Respiratory Syndrome Target a Dominant Site in the S2 Domain of the Surface Spike Glycoprotein. J. Virol..

[14] Wrapp D., Wang N., Corbett K.S., Goldsmith J.A., Hsieh C.-L., Abiona O., Graham B.S., McLellan J.S. (2020). Cryo-EM structure of the 2019-nCoV spike in the prefusion conformation. Science.

[15] Walls A.C., Park Y.J., Tortorici M.A., Wall A., McGuire A.T., Veesler D. (2020). Structure, Function, and Antigenicity of the SARS-CoV-2 Spike Glycoprotein. Cell.

[16] Hoffmann M., Kleine-Weber H., Schroeder S., Krüger N., Herrler T., Erichsen S., Schiergens T.S., Herrler G., Wu N.-H., Nitsche A. (2020). SARS-CoV-2 Cell Entry Depends on ACE2 and TMPRSS2 and Is Blocked by a Clinically Proven Protease Inhibitor. Cell.

[17] Ul Qamar M.T., Saleem S., Ashfaq U.A., Bari A., Anwar F., Alqahtani S. (2019). Epitope-based peptide vaccine design and target site depiction against Middle East Respiratory Syndrome Coronavirus: An immune-informatics study. J. Transl. Med..

[18] Shang J., Ye G., Shi K., Wan Y., Luo C., Aihara H., Geng Q., Auerbach A., Li F. (2020). Structural basis of receptor recognition by SARS-CoV-2. Nature.

[19] Lan J., Ge J., Yu J., Shan S., Zhou H., Fan S., Zhang Q., Shi X., Wang Q., Zhang L. (2020). Structure of the SARS-CoV-2 spike receptor-binding domain bound to the ACE2 receptor. Nature.

[20] Yan R., Zhang Y., Li Y., Xia L., Guo Y., Zhou Q. (2020). Structural basis for the recognition of SARS-CoV-2 by full-length human ACE2. Science.

[21] Wang Q., Zhang Y., Wu L., Niu S., Song C., Zhang Z., Lu G., Qiao C., Hu Y., Yuen K.Y. (2020). Structural and Functional Basis of SARS-CoV-2 Entry by Using Human ACE2. Cell.

[22] Doytchinova I.A., Flower D.R. (2007). VaxiJen: A server for prediction of protective antigens, tumour antigens and subunit vaccines. BMC Bioinform..

[23] Weiskopf D., Angelo M.A., Azeredo E.L.D., Sidney J., Sette A. (2013). Comprehensive analysis of dengue virus-specific responses supports an HLA-linked protective role for CD8(+) T cells. Proc. Natl. Acad. Sci. USA.

[24] Paul S., Arlehamn C.S.L., Scriba T.J., Dillon M.B.C., Oseroff C., Hinz D., McKinney D.M., Pro S.C., Sidney J., Peters B. (2015). Development and validation of a broad scheme for prediction of HLA class II restricted T cell epitopes. J. Immunol. Methods.

[25] Alexis L., Pierre T., Julien R., Marek V., Philippe D., Pierre T.J.N.A.R. (2016). PEP-FOLD3: Faster de novo structure prediction for linear peptides in solution and in complex. Nucleic Acids Res..

[26] Schneidman-Duhovny D., Inbar Y., Nussinov R., Wolfson H.J. (2005). PatchDock and SymmDock: Servers for rigid and symmetric docking. Nucleic Acids Res..

[27] Andrusier N., Nussinov R., Wolfson H.J. (2007). FireDock: Fast Interaction Refinement in molecular docking. Proteins Struct. Funct. Bioinform..

[28] Efrat M., Dina S.D., Nelly A., Ruth N., Wolfson H.J. (2008). FireDock: A web server for fast interaction refinement in molecular docking. Nucleic Acids Res..

[29] Sui J., Li W., Murakami A., Tamin A., Matthews L.J., Wong S.K., Moore M.J., Tallarico A.S.C., Olurinde M., Choe H. (2004). Potent neutralization of severe acute respiratory syndrome (SARS) coronavirus by a human mAb to S1 protein that blocks receptor association. Proc. Natl. Acad. Sci. USA.

[30] Zhu Z., Chakraborti S., He Y., Roberts A., Sheahan T., Xiao X., Hensley L.E., Prabakaran P., Rockx B., Sidorov I.A. (2007). Potent cross-reactive neutralization of SARS coronavirus isolates by human monoclonal antibodies. Proc. Natl. Acad. Sci. USA.

[31] Lv Y., Ruan Z., Wang L., Ni B., Wu Y. (2009). Identification of a novel conserved HLA-A*0201-restricted epitope from the spike protein of SARS-CoV. BMC Immunol..

[32] Tsao Y.P., Lin J.Y., Jan J.T., Leng C.H., Chen S.L. (2006). HLA-A*0201 T-cell epitopes in severe acute respiratory syndrome (SARS) coronavirus nucleocapsid and spike proteins. Biochem. Biophys. Res. Commun..

[33] Wang B., Chen H., Jiang X., Zhang M., Cao X. (2004). Identification of an HLA-A*0201-restricted CD8+ T-cell epitope Ssp-1 of SARS-CoV spike protein. Blood.

[34] Yang J., Eddie J., Michelle R., Laurie H., Gebe J.A., Kwok W.W. (2008). Searching immunodominant epitopes prior to epidemic: HLA class II-restricted SARS-CoV spike protein epitopes in unexposed individuals. Int. Immunol..

[35] Li F., Li W., Farzan M., Harrison S. (2005). Structure of SARS coronavirus spike receptor-binding domain complexed with receptor. Science.

[36] Du L., He Y., Zhou Y., Liu S., Zheng B., Jiang S. (2009). The spike protein of SARS-CoV—A target for vaccine and therapeutic development. Nat. Rev. Microbiol..

[37] Bisht H., Roberts A., Vogel L., Bukreyev A., Moss B. (2004). Severe acute respiratory syndrome coronavirus spike protein expressed by attenuated vaccinia virus protectively immunizes mice. Proc. Natl. Acad. Sci. USA.

[38] Czub M., Weingartl H., Czub S., He R., Cao J. (2005). Evaluation of modified vaccinia virus Ankara based recombinant SARS vaccine in ferrets. Vaccine.

[39] He Y., Zhou Y.H., Luo B., Chen J., Li W., Jiang S. (2004). Identification of immunodominant sites on the spike protein of severe acute respiratory syndrome (SARS) coronavirus: Implication for developing SARS diagnostics and vaccines. J. Immunol..

[40] Zhao J., Wang W., Yuan Z., Jia R., Zhao Z., Xu X., Lv P., Zhang Y., Jiang C., Gao X.M. (2007). A study on antigenicity and receptor-binding ability of fragment 450–650 of the spike protein of SARS coronavirus. Virology.

[41] Zhang H., Wang G., Li J., Nie Y., Shi X., Lian G., Wang W., Yin X., Zhao Y., Qu X. (2004). Identification of an Antigenic Determinant on the S2 Domain of the Severe Acute Respiratory Syndrome Coronavirus Spike Glycoprotein Capable of Inducing Neutralizing Antibodies. J. Virol..

[42] Hu H., Li L., Kao R.Y., Kou B., Wang Z., Zhang L., Zhang H., Hao Z., Tsui W.H., Ni A. (2005). Screening and Identification of Linear B-Cell Epitopes and Entry-Blocking Peptide of Severe Acute Respiratory Syndrome (SARS)-Associated Coronavirus Using Synthetic Overlapping Peptide Library. J. Comb. Chem..

[43] Walls A.C., Xiong X., Park Y.J., Tortorici M.A., Snijder J., Quispe J., Cameroni E., Gopal R., Dai M., Lanzavecchia A. (2019). Unexpected Receptor Functional Mimicry Elucidates Activation of Coronavirus Fusion. Cell.

[44] Poh C.M., Carissimo G., Wang B., Amrun S.N., Lee C.Y., Chee R.S., Fong S.W., Yeo N.K., Lee W.H., Torres-Ruesta A. (2020). Two linear epitopes on the SARS-CoV-2 spike protein that elicit neutralising antibodies in COVID-19 patients. Nat. Commun..

[45] Hwang W.C., Lin Y., Santelli E., Sui J., Jaroszewski L., Stec B., Farzan M., Marasco W.A., Liddington R.C. (2006). Structural Basis of Neutralization by a Human Anti-severe Acute Respiratory Syndrome Spike Protein Antibody, 80R. J. Biol. Chem..

[46] Prabakaran P., Gan J., Feng Y., Zhu Z., Choudhry V., Xiao X., Ji X., Dimitrov D.S. (2006). Structure of Severe Acute Respiratory Syndrome Coronavirus Receptor-binding Domain Complexed with Neutralizing Antibody. J. Biol. Chem..

[47] De Groot A.S., Moise L., Terry F., Gutierrez A.H., Hindocha P., Richard G., Hoft D.F., Ross T.M., Noe A.R., Takahashi Y. (2020). Better Epitope Discovery, Precision Immune Engineering, and Accelerated Vaccine Design Using Immunoinformatics Tools. Front. Immunol..

[48] Gutiérrez A.H., Loving C., Moise L., Terry F.E., Brockmeier S.L., Hughes H.R., Martin W.D., De Groot A.S. (2016). In Vivo Validation of Predicted and Conserved T Cell Epitopes in a Swine Influenza Model. PLoS ONE.

[49] Hewitt J.S., Karuppannan A.K., Tan S., Gauger P., Halbur P.G., Gerber P.F., De Groot A.S., Moise L., Opriessnig T. (2019). A prime-boost concept using a T-cell epitope-driven DNA vaccine followed by a whole virus vaccine effectively protected pigs in the pandemic H1N1 pig challenge model. Vaccine.

[50] Song P., Li W., Xie J., Hou Y., You C. (2020). Cytokine Storm Induced by SARS-CoV-2. Clin. Chim. Acta Int. J. Clin. Chem..

[51] Graham R.L., Donaldson E.F., Baric R.S. (2013). A decade after SARS: Strategies for controlling emerging coronaviruses. Nat. Rev. Microbiol..

[52] Yong C.Y., Ong H.K., Yeap S.K., Ho K.L., Tan W.S. (2019). Recent Advances in the Vaccine Development Against Middle East Respiratory Syndrome-Coronavirus. Front. Microbiol..

