# Supplementary material for: Immunoinformatic Analysis of T- and B-Cell Epitopes for SARS-CoV-2 Vaccine Design
Source: Vaccines (Basel). 2020 Jul 3;8(3):355. doi: 10.3390/vaccines8030355 (PMC7563688; doi:10.3390/vaccines8030355)
Supplement: Supplementary file 1 [file vaccines-08-00355-s001.zip › vaccines-833738-supplementary figures-for proof.docx]

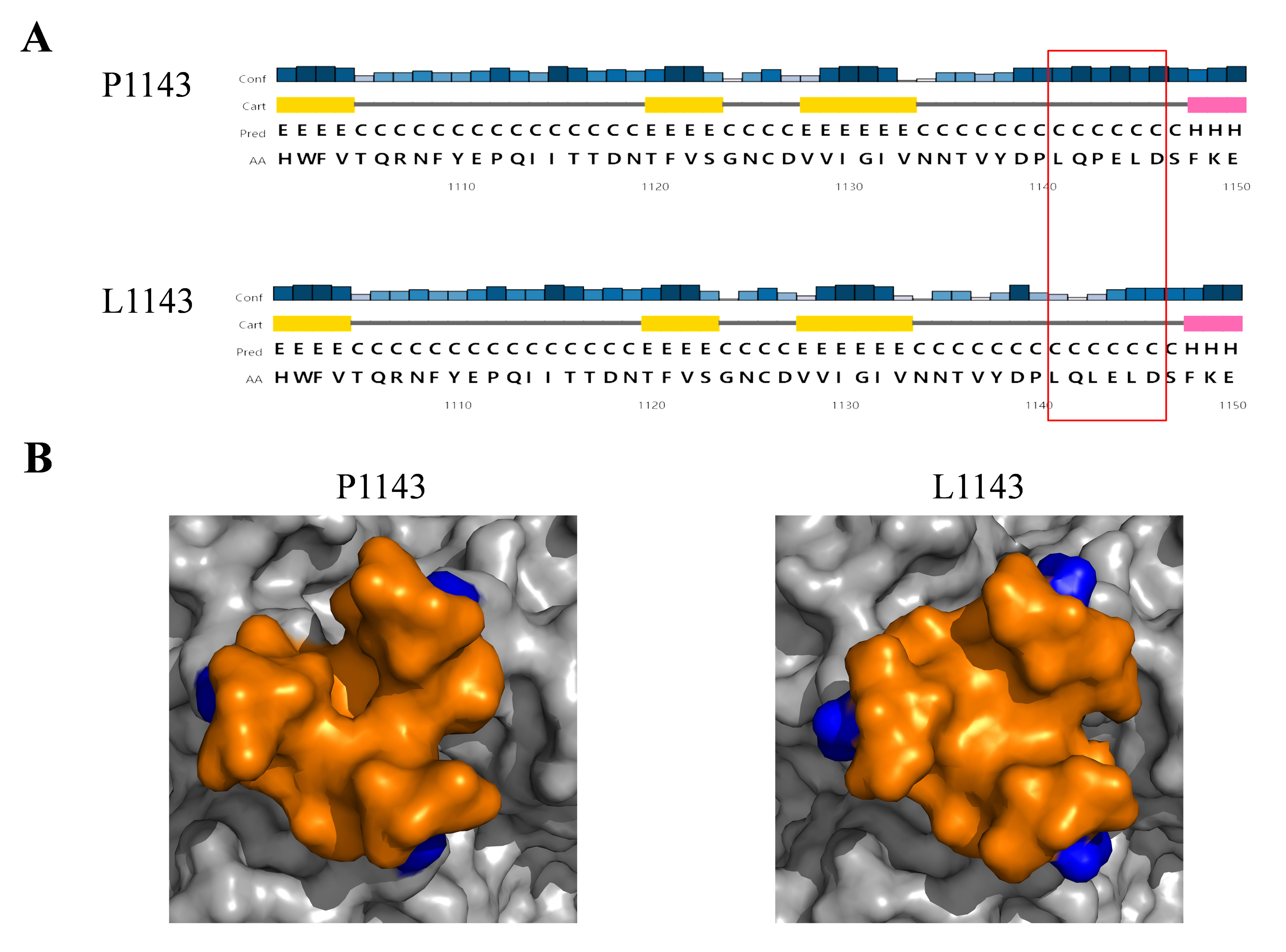


**Figure S1.** The effect of P1143L mutation on the secondary structure (A) and predicted 3D structure (B). The residues 1143 is shown as blue.


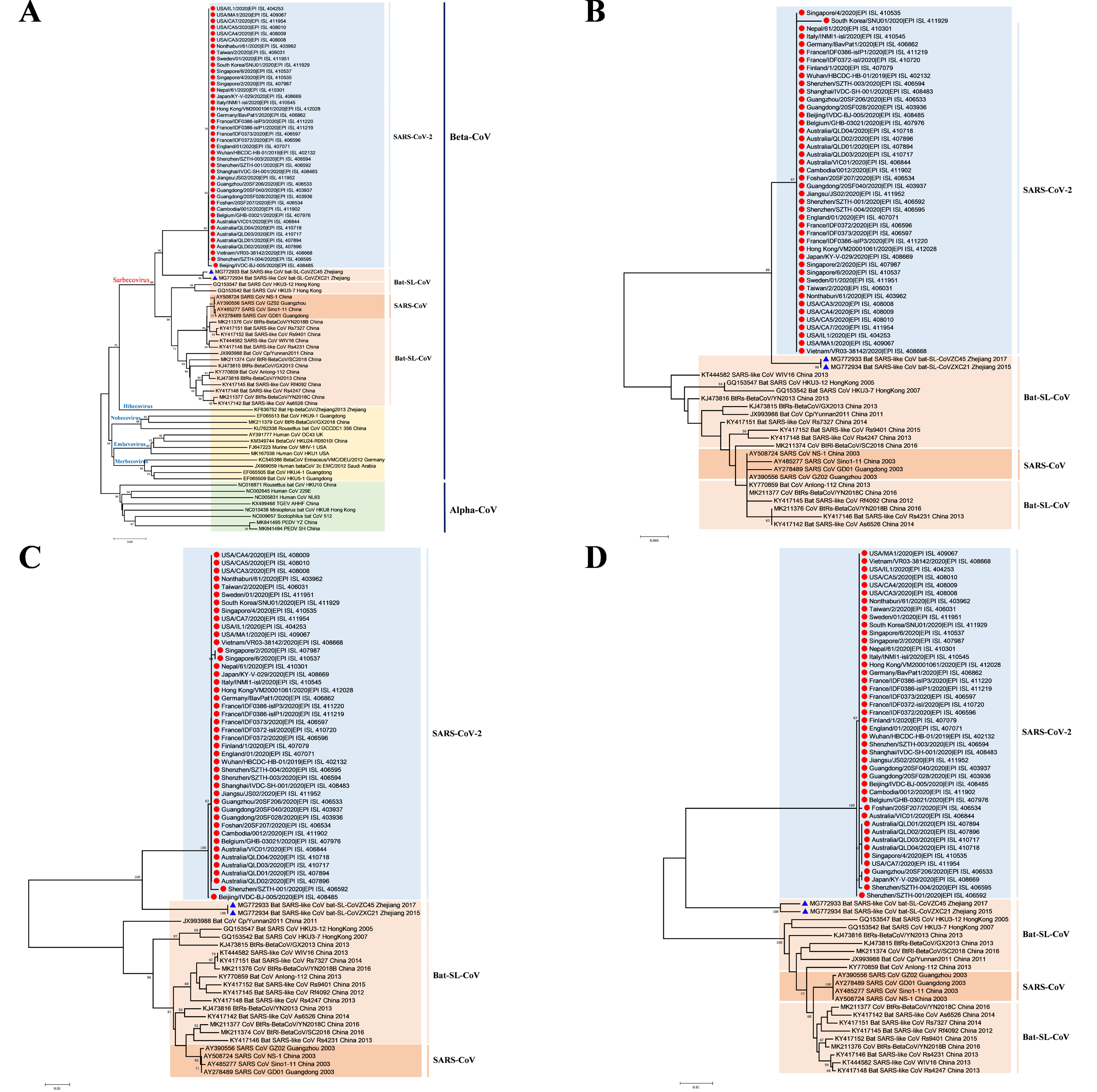


**Figure S2.** Neighbor joining tree analysis of SARS-CoV-2. The trees were constructed by the neighbor joining (NJ) phylogenetic method using the *p*-distance model with 1000 bootstrap replication and bootstrap >50% are shown. A to D correspond to the *s, e, m* and *n* gene, respectively.


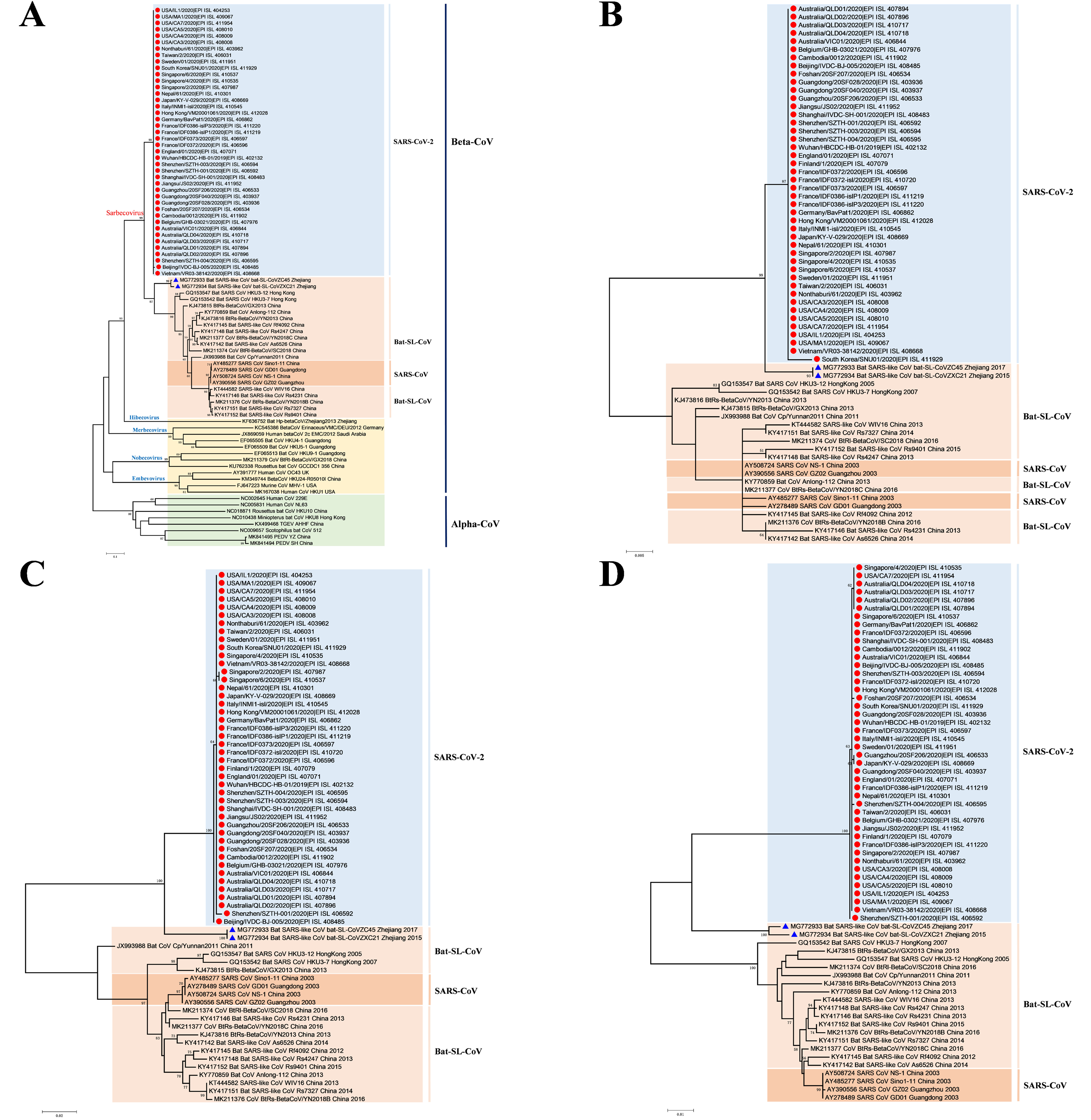


**Figure S3.** Maximum-likelihood tree analysis of SARS-CoV-2. The trees were constructed by the Maximum-likelihood (ML) phylogenetic method using the GTR model with 1000 bootstrap replication and bootstrap >50% are shown. A to D correspond to the *s, e, m* and *n* gene, respectively.


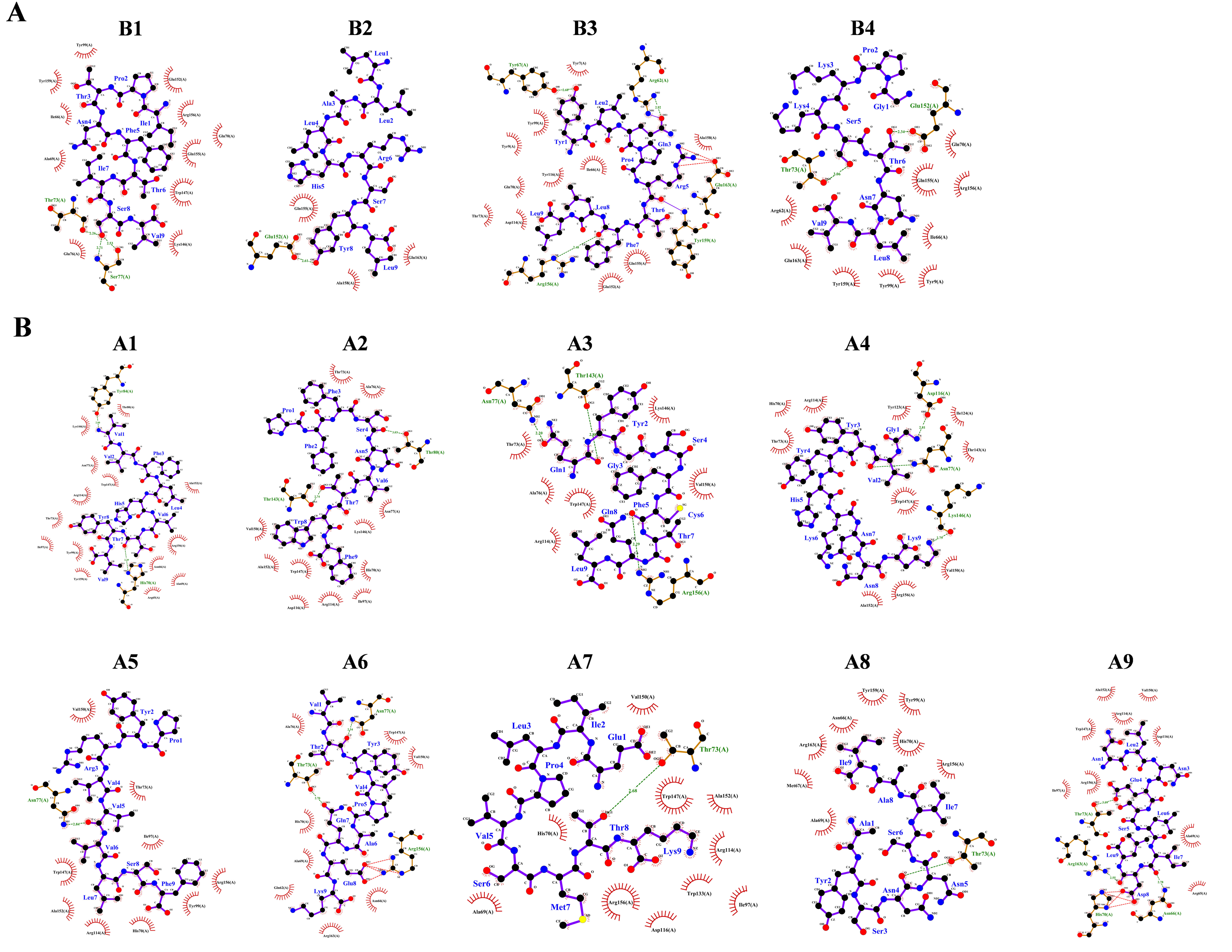


**Figure S4.** (**A**) 2D graphical representation of interaction analyses between human HLA-B7 protein and MHC-I binding peptides. B1 to B4 correspond to the peptides with information provided in Table 4 and Figure 3A. The images showing the residues interacting with strong hydrogen bonds. (**B**) 2D graphical representation of interaction analyses between human HLA-A*01:01 protein and MHC-I binding peptides. A1 to A9 correspond to the peptides with information provided in Table 4 and Figure 3B. The images showing the residues interacting with strong hydrogen bonds.


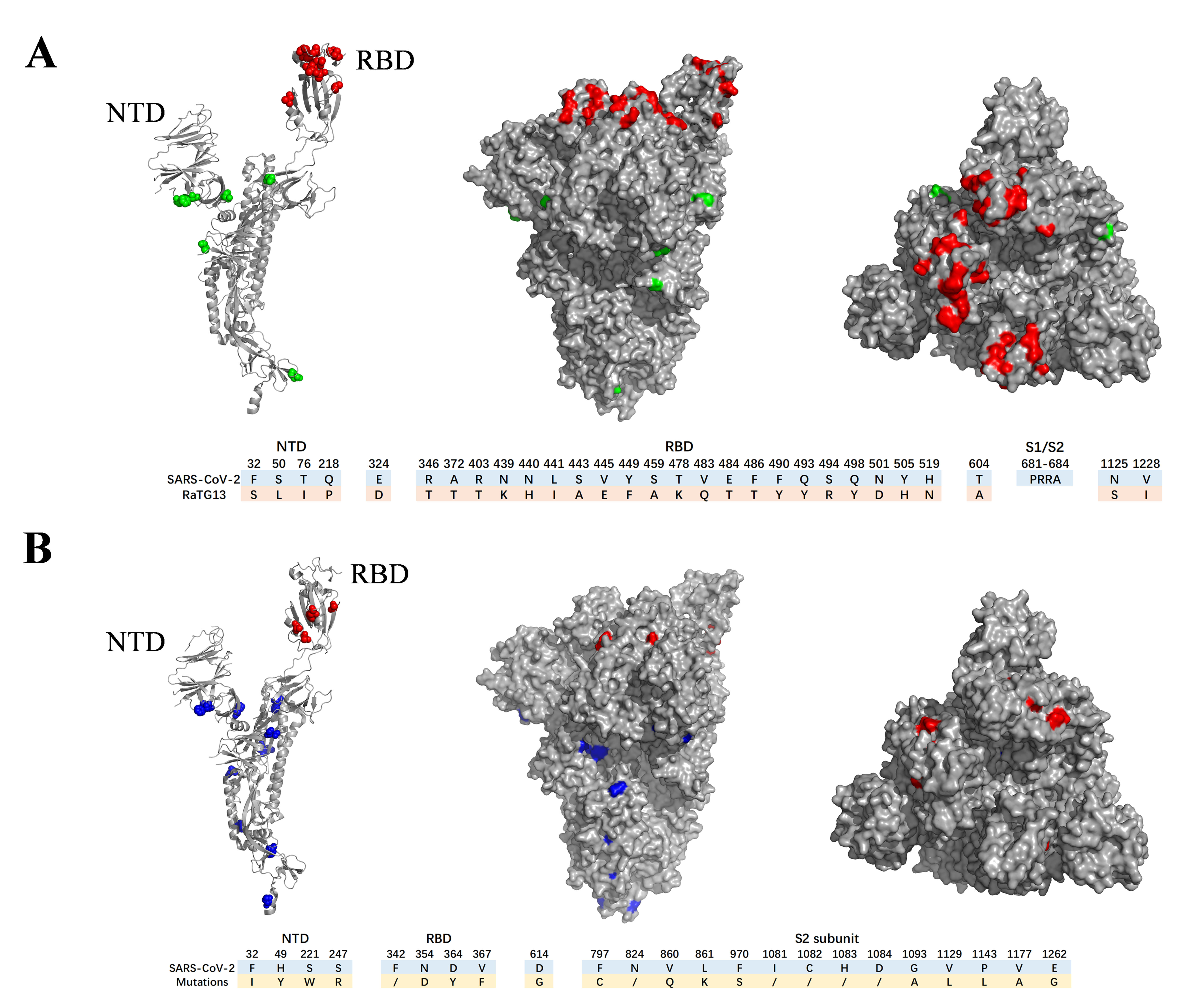


**Figure S5.** (**A**) Sequence variability of S protein between RaTG13 and SARS-CoV-2. RaTG13 variant residues in RBD are shown as red spheres, others are shown as green spheres. (**B**) Sequence variability of S protein among 138 SARS-CoV-2 clinical isolates. SARS-CoV-2 variant residues in RBD are shown as red spheres, others are shown as blue spheres.
