# Supplementary material for: Immunoinformatic Analysis of T- and B-Cell Epitopes for SARS-CoV-2 Vaccine Design
Source: Vaccines (Basel). 2020 Jul 3;8(3):355. doi: 10.3390/vaccines8030355 (PMC7563688; doi:10.3390/vaccines8030355)
Supplement: Supplementary file 1 [file vaccines-08-00355-s001.zip › vaccines-833738-supplementary tables-for proof.docx]

**Table S1.** 17 predicted linear B cell epitopes of SARS-CoV-2 S protein using BepiPred 2.0.

| **Position** | **Peptide** | **Length** | **Vaxijen Score** |
| --- | --- | --- | --- |
| 15–31 | CVNLTTRTQLPPAYTNS | 17 | 1.2219 |
| 62–75 | VTWFHAIHVSGTNG | 14 | 0.5786 |
| 141–152 | LGVYYHKNNKSW | 13 | 0.8156 |
| 208–220 | TPINLVRDLPQGF | 13 | 0.4768 |
| 252–259 | GDSSSGWT | 8 | 0.2201 |
| 341–361 | VFNATRFASVYAWNRKRISNC | 21 | 0.2783 |
| 405–418 | DEVRQIAPGQTGKI | 14 | 0.9312 |
| 441–448 | LDSKVGGN | 8 | 0.8773 |
| 469–478 | STEIYQAGST | 10 | 0.0944 |
| 525–533 | CGPKKSTNL | 9 | 0.1363 |
| 657–664 | NNSYECDI | 8 | 0.6539 |
| 674–688 | YQTQTNSPRRARSVA | 15 | 0.2617 |
| 696–709 | TMSLGAENSVAYSN | 14 | 0.6780 |
| 789–798 | YKTPPIKDFG | 10 | 0.0200 |
| 1136–1143 | TVYDPLQP | 8 | 0.3135 |
| 1154–1169 | KYFKNHTSPDVDLGDI | 16 | 0.7333 |
| 1 256–1263 | FDEDDSEP | 8 | 0.3300 |

**Table S2.** Linear B cell epitopes of SARS-CoV S protein.

| **Epitope ID** | **SARS-CoV Sequence** | **Start** | **End** | **Identity (87 SARS-CoV Isolates)** | **Response Frequency** | **Neutralizing Activities** |
| --- | --- | --- | --- | --- | --- | --- |
| 7066 | CSQNPLAELKCSVKSFEIDKGIYQTSNFRVVPSGD | 278 | 312 | 95.40% | 0.95 | Non tested |
| 6334 | CGPKLSTDLIKNQCVNFNFNGLTGTGVLTPSSKRFQPFQQFGRDVSDFTD | 511 | 560 | 100% | 1 | Non tested |
| 71191 | VSVITPGTNASSEVAVLYQDVNCTDVSTAIHADQLTPAWRIYSTGNNVFQ | 581 | 630 | 43.18% | 1 | Non tested |
| 51097 | QILPDPLKPTKRSFIEDLLFNKVTLA | 786 | 811 | 93.10% | 0.58 | Yes |
| 47479 | PFAMQMAYRFNGIGVTQ | 879 | 895 | 100% | 0.57 | Non tested |
| 46822 | PAICHEGKAYFPREGVFVFNGTSWFITQRNFFS | 1061 | 1093 | 96.55% | 0.8 | No |
| 12426 | EIDRLNEVAKNLNESLIDLQELGKYEQY | 1164 | 1191 | 95.40% | 0.67 | Non tested |

**Table S3.** Discontinuous B cell epitopes of SARS-CoV S protein.

| **Epitope ID** | **mAb** | **Sequence** |
| --- | --- | --- |
| 77444 | m396 | T359, T363, K365, K390, G391, D392, R395, R426, Y436, G482, Y484, T485, T486, T487, G488, I489, G490, Y491, Q492, Y494 |
| 910052 | S230 | G446, P462, D463, Y475 |
| 77442 | 80R | R426, S432, T433, Y436, N437, K439, Y440, Y442, P469, P470, A471, L472, N473, C474, Y475, W476, L478, N479, D480, Y481, G482, Y484, T485, T486, T487, G488, I489, Y491, Q492 |

**Table S4.** Predicted MHC class-I epitopes in SARS-CoV-2 S protein.

| **Start** | **End** | **Length** | **Peptide** | **Alleles (Percentile Rank)** | **Vaxijen Score** |
| --- | --- | --- | --- | --- | --- |
| 57 | 65 | 9 | PFFSNVTWF | HLA-A*24:02 (0.07) | 0.6638 |
| 89 | 97 | 9 | GVYFASTEK | HLA-A*03:01 (0.01), HLA-A*11:01 (0.01) | 0.7112 |
| 109 | 117 | 9 | TLDSKTQSL | HLA-B*08:01 (0.14), HLA-A*01:01 (1), HLA-B*07:02 (1.5) | 1.0685 |
| 142 | 150 | 9 | GVYYHKNNK | HLA-A*03:01 (0.03), HLA-A*11:01 (0.12) | 0.8264 |
| 168 | 176 | 9 | FEYVSQPFL | HLA-B*40:01 (0.04) | 0.6324 |
| 241 | 249 | 9 | LLALHRSYL | HLA-B*08:01 (0.14), HLA-A*02:01 (1.5), HLA-B*07:02 (1.9) | 0.5241 |
| 258 | 266 | 9 | WTAGAAAYY | HLA-A*01:01 (0.03), HLA-A*11:01 (0.73), HLA-A*03:01 (1.4) | 0.6306 |
| 269 | 277 | 9 | YLQPRTFLL | HLA-A*02:01 (0.02), HLA-B*08:01 (0.03), HLA-A*24:02 (0.35), HLA-A*01:01 (0.9), HLA-B*07:02 (1.8) | 0.4532 |
| 292 | 300 | 9 | ALDPLSETK | HLA-A*11:01 (0.16), HLA-A*03:01 (0.29), HLA-A*01:01 (1.5) | 1.2241 |
| 311 | 319 | 9 | GIYQTSNFR | HLA-A*03:01 (0.2), HLA-A*11:01 (0.24) | 0.538 |
| 349 | 357 | 9 | SVYAWNRKR | HLA-A*03:01 (0.2), HLA-A*11:01 (0.25) | 0.765 |
| 378 | 386 | 9 | KCYGVSPTK | HLA-A*03:01 (0.25), HLA-A*11:01 (0.81) | 1.4199 |
| 409 | 417 | 9 | QIAPGQTGK | HLA-A*03:01 (0.14), HLA-A*11:01 (0.16) | 1.8297 |
| 417 | 425 | 9 | KIADYNYKL | HLA-A*02:01 (0.04), HLA-A*24:02 (1.5), HLA-B*08:01 (1.7), HLA-B*07:02 (1.6) | 1.6639 |
| 489 | 497 | 9 | YFPLQSYGF | HLA-A*24:02 (0.03) | 0.5107 |
| 507 | 515 | 9 | PYRVVVLSF | HLA-A*24:02 (0.1) | 1.0281 |
| 526 | 534 | 9 | GPKKSTNLV | HLA-B*07:02 (0.15), HLA-B*08:01 (1.3) | 0.6828 |
| 550 | 558 | 9 | GVLTESNKK | HLA-A*11:01 (0.12), HLA-A*03:01 (0.34) | 0.8797 |
| 604 | 612 | 9 | TSNQVAVLY | HLA-A*01:01 (0.04), HLA-A*11:01 (0.52), HLA-A*03:01 (1.1) | 0.4387 |
| 652 | 660 | 9 | GAEHVNNSY | HLA-A*01:01 (0.14) | 0.9347 |
| 680 | 688 | 9 | SPRRARSVA | HLA-B*07:02 (0.08), HLA-B*08:01 (1.2) | 0.7729 |
| 691 | 699 | 9 | SIIAYTMSL | HLA-A*02:01 (0.11), HLA-B*08:01 (0.25), HLA-B*07:02 (0.61) | 0.5234 |
| 706 | 714 | 9 | AYSNNSIAI | HLA-A*24:02 (0.17) | 0.8274 |
| 714 | 722 | 9 | IPTNFTISV | HLA-B*07:02 (0.11) | 0.882 |
| 725 | 733 | 9 | EILPVSMTK | HLA-A*11:01 (0.09), HLA-A*03:01 (0.24) | 1.6842 |
| 733 | 741 | 9 | KTSVDCTMY | HLA-A*01:01 (0.28), HLA-A*11:01 (1.4), HLA-A*03:01 (2) | 1.1824 |
| 755 | 763 | 9 | QYGSFCTQL | HLA-A*24:02 (0.31) | 1.2906 |
| 821 | 829 | 9 | LLFNKVTLA | HLA-A*02:01 (0.12), HLA-B*08:01 (1.8) | 0.615 |
| 827 | 835 | 9 | TLADAGFIK | HLA-A*11:01 (0.05), HLA-A*03:01 (0.11) | 0.5781 |
| 857 | 865 | 9 | GLTVLPPLL | HLA-A*02:01 (0.2) | 0.6621 |
| 939 | 947 | 9 | SSTASALGK | HLA-A*11:01 (0.09), HLA-A*03:01 (0.31) | 0.6215 |
| 996 | 1004 | 9 | LITGRLQSL | HLA-B*08:01 (0.13), HLA-B*07:02 (1.6) | 0.8238 |
| 1020 | 1028 | 9 | ASANLAATK | HLA-A*11:01 (0.01), HLA-A*03:01 (0.07) | 0.7014 |
| 1060 | 1068 | 9 | VVFLHVTYV | HLA-A*02:01 (0.19), HLA-B*08:01 (1.4) | 1.5122 |
| 1065 | 1073 | 9 | VTYVPAQEK | HLA-A*11:01 (0.01), HLA-A*03:01 (0.02) | 0.8132 |
| 1101 | 1109 | 9 | HWFVTQRNF | HLA-A*24:02 (0.14) | 0.746 |
| 1181 | 1189 | 9 | KEIDRLNEV | HLA-B*40:01 (0.09), HLA-A*02:01 (1.9) | 0.53 |
| 1192 | 1200 | 9 | NLNESLIDL | HLA-A*02:01 (0.19), HLA-B*08:01 (1.9) | 0.6827 |
| 1208 | 1216 | 9 | QYIKWPWYI | HLA-A*24:02 (0.02) | 1.4177 |
| 1262 | 1270 | 9 | EPVLKGVKL | HLA-B*08:01 (0.13), HLA-B*07:02 (0.18) | 1.2301 |

**Table S5.** MHC class I and Class II epitopes identified in SARS-CoV S protein.

| **Epitope ID** | **Sequence** | **Start** | **End** | **Response Frequency** | **MHC class** |
| --- | --- | --- | --- | --- | --- |
| 32069 | KLPDDFMGCV | 411 | 420 | 1 | Class I |
| 50166  58730 | PYRVVVLSF  SIVAYTMSL | 493  673 | 501  681 | Not available  1 |  |
| 27241 | ILPDPLKPT | 787 | 795 | 1 |  |
| 2801 | ALNTLVKQL | 940 | 948 | 1 |  |
| 69657 | VLNDILSRL | 958 | 966 | 1 |  |
| 36724 | LITGRLQSL | 978 | 986 | 1 |  |
| 44814 | NLNESLIDL | 1174 | 1182 | 1 |  |
| 16156 | FIAGLIAIV | 1202 | 1210 | 1 |  |
|  | | | | | |
| 99995 | EIFRSDTLYLTQDLFLPF | 45 | 62 | 1 | Class II |
| 100665 | YLTQDLFLPFYSNVTGFH | 53 | 70 | 1 |  |
| 100553 | TGFHTINHTFGNPVIPFK | 67 | 84 | 1 |  |
| 100347 | NPFFAVSKPMGTQTHTMI | 135 | 152 | 1 |  |
| 100316 | NAFNCTFEYISDAFSLDV | 155 | 172 | 1 |  |
| 99926 | DAFSLDVSEKSGN | 166 | 178 | 0.75 |  |
| 100561 | TLKPIFKLPLGINITNFR | 215 | 232 | 1 |  |
| 100567 | TNFRAILTAFSPAQDIW | 229 | 245 | 0.67 |  |
| 100541 | TAFSPAQDIWGTSAAAYF | 236 | 253 | 1 |  |
| 33305 | KSFEIDKGIYQTSNFRVV | 291 | 308 | 0.67 |  |
| 29666 | IYQTSNFRVVPSGDVVRF | 299 | 316 | 1 |  |
| 61598 | STFFSTFKCYGVSATKL | 358 | 374 | 1 |  |
| 44199 | NIDATSTGNYNYKYRYLR | 427 | 444 | 1 |  |
| 100664 | YLRHGKLRPFERDISNVP | 442 | 459 | 0.5 |  |
| 100481 | RPFERDISNVPFS | 449 | 461 | 0.5 |  |
| 37758 | LLRSTSQKSIVAYTMSL | 665 | 681 | 1 |  |
| 63309 | TECANLLLQYGSFCTQL | 729 | 745 | 1 |  |
| 100612 | VKQMYKTPTLKYFGGFNF | 767 | 784 | 0.67 |  |
| 99899 | AQKFNGLTVLPPLLTDDM | 834 | 851 | 1 |  |
| 100048 | GAALQIPFAMQMAYRF | 873 | 888 | 1 |  |
| 14208 | ESLTTTSTALGKLQDVV | 918 | 934 | 1 |  |
| 100428 | QLIRAAEIRASANLAATK | 993 | 1010 | 0.5 |  |
| 100537 | SWFITQRNFFSPQII | 1083 | 1097 | 0.67 |  |

Peptides that are identical in the SARS-CoV-2 sequences are underlined.

**Table S6.** Detailed information of 138 SARS-CoV-2 sequences.

| **Accession ID** | **Virus Name** | **Location** | **Collection Date** | **Host** | **Specimen Source** |
| --- | --- | --- | --- | --- | --- |
| EPI_ISL_406844 | BetaCoV/Australia/VIC01/2020 | Australia | 2020/1/25 | Human |  |
| EPI_ISL_407893 | BetaCoV/Australia/NSW01/2020 | Australia | 2020/1/24 | Human | Nasopharyngeal swab |
| EPI_ISL_407894 | BetaCoV/Australia/QLD01/2020 | Australia | 2020/1/28 | Human | Pharyngeal swab |
| EPI_ISL_407896 | BetaCoV/Australia/QLD02/2020 | Australia | 2020/1/30 | Human | Aspirate |
| EPI_ISL_408976 | BetaCoV/Sydney/2/2020 | Australia | 2020/1/22 | Human | Nasopharyngeal swab |
| EPI_ISL_408977 | BetaCoV/Sydney/3/2020 | Australia | 2020/1/25 | Human | Nasopharyngeal swab |
| EPI_ISL_410717 | BetaCoV/Australia/QLD03/2020 | Australia | 2020/2/5 | Human | Nasopharyngeal swab |
| EPI_ISL_410718 | BetaCoV/Australia/QLD04/2020 | Australia | 2020/2/5 | Human | Nasopharyngeal swab |
| EPI_ISL_407976 | BetaCoV/Belgium/GHB-03021/2020 | Belgium | 2020/2/3 | Human | Oropharyngeal swab |
| EPI_ISL_411902 | BetaCoV/Cambodia/0012/2020 | Cambodia | 2020/1/27 | Human | Nasopharyngeal swab and oropharyngeal swab |
| EPI_ISL_402119 | BetaCoV/Wuhan/IVDC-HB-01/2019 | China-Wuhan | 2019/12/30 | Human | Alveolar lavage fluid |
| EPI_ISL_402120 | BetaCoV/Wuhan/IVDC-HB-04/2020 | China-Wuhan | 2020/1/1 | Human | Alveolar lavage fluid |
| EPI_ISL_402121 | BetaCoV/Wuhan/IVDC-HB-05/2019 | China-Wuhan | 2019/12/30 | Human | Alveolar lavage fluid |
| EPI_ISL_402123 | BetaCoV/Wuhan/IPBCAMS-WH-01/2019 | China-Wuhan | 2019/12/24 | Human | Bronchoalveolar lavage fluid |
| EPI_ISL_402124 | BetaCoV/Wuhan/WIV04/2019 | China-Wuhan | 2019/12/30 | Human | Bronchoalveolar lavage fluid |
| EPI_ISL_402125 | BetaCoV/Wuhan-Hu-1/2019 | China | 2019/12/31 | Human | Bronchoalveolar lavage fluid |
| EPI_ISL_402127 | BetaCoV/Wuhan/WIV02/2019 | China-Wuhan | 2019/12/30 | Human | Bronchoalveolar lavage fluid |
| EPI_ISL_402128 | BetaCoV/Wuhan/WIV05/2019 | China-Wuhan | 2019/12/30 | Human | Bronchoalveolar lavage fluid |
| EPI_ISL_402129 | BetaCoV/Wuhan/WIV06/2019 | China-Wuhan | 2019/12/30 | Human | Bronchoalveolar lavage fluid |
| EPI_ISL_402130 | BetaCoV/Wuhan/WIV07/2019 | China-Wuhan | 2019/12/30 | Human | Bronchoalveolar lavage fluid |
| EPI_ISL_402132 | BetaCoV/Wuhan/HBCDC-HB-01/2019 | China-Wuhan | 2019/12/30 | Human | Alveolar lavage fluid |
| EPI_ISL_403928 | BetaCoV/Wuhan/IPBCAMS-WH-05/2020 | China-Wuhan | 2020/1/1 | Human | Bronchoalveolar lavage fluid |
| EPI_ISL_403929 | BetaCoV/Wuhan/IPBCAMS-WH-04/2019 | China-Wuhan | 2019/12/30 | Human | Bronchoalveolar lavage fluid |
| EPI_ISL_403930 | BetaCoV/Wuhan/IPBCAMS-WH-03/2019 | China-Wuhan | 2019/12/30 | Human | Bronchoalveolar lavage fluid |
| EPI_ISL_403931 | BetaCoV/Wuhan/IPBCAMS-WH-02/2019 | China-Wuhan | 2019/12/30 | Human | Bronchoalveolar lavage fluid |
| EPI_ISL_403932 | BetaCoV/Guangdong/20SF012/2020 | China-Shenzhen | 2020/1/14 | Human | endotracheal aspirates |
| EPI_ISL_403933 | BetaCoV/Guangdong/20SF013/2020 | China-Shenzhen | 2020/1/15 | Human | endotracheal aspirates |
| EPI_ISL_403934 | BetaCoV/Guangdong/20SF014/2020 | China-Shenzhen | 2020/1/15 | Human | bronchoalveolar lavage fluid |
| EPI_ISL_403935 | BetaCoV/Guangdong/20SF025/2020 | China-Shenzhen | 2020/1/15 | Human | throat swab |
| EPI_ISL_403936 | BetaCoV/Guangdong/20SF028/2020 | China-Zhuhai | 2020/1/17 | Human | throat swab |
| EPI_ISL_403937 | BetaCoV/Guangdong/20SF040/2020 | China-Zhuhai | 2020/1/18 | Human | nasal swab |
| EPI_ISL_404227 | BetaCoV/Zhejiang/WZ-01/2020 | China-Zhejiang | 2020/1/16 | Human | Sputum |
| EPI_ISL_404228 | BetaCoV/Zhejiang/WZ-02/2020 | China-Zhejiang | 2020/1/17 | Human | Sputum |
| EPI_ISL_405839 | BetaCoV/Shenzhen/HKU-SZ-005/2020 | China-Shenzhen | 2020/1/11 | Human | Sputum |
| EPI_ISL_406030 | BetaCoV/Shenzhen/HKU-SZ-002/2020 | China-Shenzhen | 2020/1/10 | Human | nasopharyngeal swab |
| EPI_ISL_406533 | BetaCoV/Guangzhou/20SF206/2020 | China-Guangzhou | 2020/1/22 | Human |  |
| EPI_ISL_406534 | BetaCoV/Foshan/20SF207/2020 | China-Foshan | 2020/1/22 | Human |  |
| EPI_ISL_406535 | BetaCoV/Foshan/20SF210/2020 | China-Foshan | 2020/1/22 | Human |  |
| EPI_ISL_406536 | BetaCoV/Foshan/20SF211/2020 | China-Foshan | 2020/1/22 | Human |  |
| EPI_ISL_406538 | BetaCoV/Guangdong/20SF201/2020 | China-Guangdong | 2020/1/23 | Human |  |
| EPI_ISL_406592 | BetaCoV/Shenzhen/SZTH-001/2020 | China-Shenzhen | 2020/1/13 | Human | Alveolar lavage fluid |
| EPI_ISL_406593 | BetaCoV/Shenzhen/SZTH-002/2020 | China-Shenzhen | 2020/1/13 | Human | Alveolar lavage fluid |
| EPI_ISL_406594 | BetaCoV/Shenzhen/SZTH-003/2020 | China-Shenzhen | 2020/1/16 | Human | Alveolar lavage fluid |
| EPI_ISL_406595 | BetaCoV/Shenzhen/SZTH-004/2020 | China-Shenzhen | 2020/1/16 | Human | Alveolar lavage fluid |
| EPI_ISL_406716 | BetaCoV/China/WHU01/2020 | China-Wuhan | 2020/1/2 | Human | BALF sample of 2019 Wuhan pneumonia patient 01 |
| EPI_ISL_406717 | BetaCoV/China/WHU02/2020 | China-Wuhan | 2020/1/2 | Human | BALF sample of 2019 Wuhan pneumonia patient 02 |
| EPI_ISL_406798 | BetaCov/Wuhan/WH01/2019 | China-Wuhan | 2019/12/26 | Human | Bronchoalveolar Lavage NCIT:C51913 |
| EPI_ISL_406800 | BetaCov/Wuhan/WH03/2020 | China-Wuhan | 2020/1/1 | Human | Bronchoalveolar Lavage NCIT:C51913 |
| EPI_ISL_406801 | BetaCov/Wuhan/WH04/2020 | China-Wuhan | 2020/1/5 | Human | Bronchoalveolar Lavage NCIT:C51913 |
| EPI_ISL_406970 | BetaCoV/Hangzhou/HZ-1/2020 | China-Hangzhou | 2020/1/20 | Human | Sputum |
| EPI_ISL_407313 | BetaCoV/Hangzhou/HZCDC0001/2020 | China-Hangzhou | 2020/1/19 | Human | Sputum |
| EPI_ISL_408478 | BetaCoV/Chongqing/YC01/2020 | China-Chongqing | 2020/1/21 | Human | Nasopharyngeal swab |
| EPI_ISL_408479 | BetaCoV/Chongqing/ZX01/2020 | China-Chongqing | 2020/1/23 | Human | Nasopharyngeal swab |
| EPI_ISL_408480 | BetaCoV/Yunnan/IVDC-YN-003/2020 | China-Kunming | 2020/1/17 | Human | Sputum |
| EPI_ISL_408481 | BetaCoV/Chongqing/IVDC-CQ-001/2020 | China-Chongqing | 2020/1/18 | Human | Throat swab |
| EPI_ISL_408482 | BetaCoV/Shandong/IVDC-SD-001/2020 | China-Qingdao | 2020/1/19 | Human | Sputum |
| EPI_ISL_408483 | BetaCoV/Shanghai/IVDC-SH-001/2020 | China-Shanghai | 2020/1/20 | Human | Bronchoalveolar lavage fluid |
| EPI_ISL_408484 | BetaCoV/Sichuan/IVDC-SC-001/2020 | China-Chengdu | 2020/1/15 | Human | Bronchoalveolar lavage fluid |
| EPI_ISL_408485 | BetaCoV/Beijing/IVDC-BJ-005/2020 | China-Beijing | 2020/1/18 | Human | Throat swab |
| EPI_ISL_408486 | BetaCoV/Jiangxi/IVDC-JX-002/2020 | China-Pingxiang | 2020/1/11 | Human | Sputum |
| EPI_ISL_408488 | BetaCoV/Jiangsu/IVDC-JS-001/2020 | China-Huaian | 2020/1/19 | Human | Alveolar lavage fluid |
| EPI_ISL_408514 | BetaCoV/Wuhan/IVDC-HB-envF13-20/2020 | China-Wuhan | 2020/1/1 | Environment |  |
| EPI_ISL_408515 | BetaCoV/Wuhan/IVDC-HB-envF13-21/2020 | China-Wuhan | 2020/1/1 | Environment |  |
| EPI_ISL_411060 | BetaCoV/Fujian/8/2020 | China-Fujian | 2020/1/21 | Human |  |
| EPI_ISL_411066 | BetaCoV/Fujian/13/2020 | China-Fujian | 2020/1/22 | Human |  |
| EPI_ISL_411950 | BetaCoV/Jiangsu/JS01/2020 | China-Jiangsu | 2020/1/23 | Human |  |
| EPI_ISL_411952 | BetaCoV/Jiangsu/JS02/2020 | China-Jiangsu | 2020/1/24 | Human |  |
| EPI_ISL_411953 | BetaCoV/Jiangsu/JS03/2020 | China-Jiangsu | 2020/1/24 | Human |  |
| EPI_ISL_411957 | BetaCoV/China/WH-09/2020 | China | 2020/1/8 | Human | Throat swab |
| EPI_ISL_412026 | BetaCoV/Hefei/2/2020 | China-Hefei | 2020/2/23 | Human | Other |
| EPI_ISL_412459 | BetaCoV/Jingzhou/HBCDC-HB-01/2020 | China-Jingzhou | 2020/1/8 | Human | Alveolar lavage fluid |
| EPI_ISL_407071 | BetaCoV/England/01/2020 | England | 2020/1/29 | Human | swab |
| EPI_ISL_407073 | BetaCoV/England/02/2020 | England | 2020/1/29 | Human | swab |
| EPI_ISL_412116 | BetaCoV/England/09c/2020 | England | 2020/2/9 | Human | Nasal swab |
| EPI_ISL_406596 | BetaCoV/France/IDF0372/2020 | France | 2020/1/23 | Human | Oro-Pharyngeal swab |
| EPI_ISL_406597 | BetaCoV/France/IDF0373/2020 | France | 2020/1/23 | Human | Oro-pharyngeal swab |
| EPI_ISL_408430 | BetaCoV/France/IDF0515/2020 | France | 2020/1/29 | Human | Oro-pharyngeal swab |
| EPI_ISL_408431 | BetaCov/France/IDF0626/2020 | France | 2020/1/29 | Human | Oro-pharyngeal swab |
| EPI_ISL_410486 | BetaCoV/France/RA739/2020 | France | 2020/2/8 | Human | Oro-pharyngeal swab |
| EPI_ISL_410720 | BetaCoV/France/IDF0372-isl/2020 | France | 2020/1/23 | Human | Oro-Pharyngeal swab |
| EPI_ISL_410984 | BetaCoV/France/IDF0515-isl/2020 | France | 2020/1/29 | Human | Oro-pharyngeal swab |
| EPI_ISL_411218 | BetaCoV/France/IDF0571/2020 | France | 2020/2/2 | Human | Nasopharyngeal swab |
| EPI_ISL_411219 | BetaCoV/France/IDF0386-islP1/2020 | France | 2020/1/28 | Human | Nasopharyngeal swab |
| EPI_ISL_411220 | BetaCoV/France/IDF0386-islP3/2020 | France | 2020/1/28 | Human | Nasopharyngeal swab |
| EPI_ISL_406862 | BetaCoV/Germany/BavPat1/2020 | Germany | 2020/1/28 | Human | Sputum |
| EPI_ISL_412028 | BetaCoV/Hong Kong/VM20001061/2020 | Hong Kong | 2020/1/22 | Human | Nasopharyngeal aspirate & Throat swab |
| EPI_ISL_412029 | BetaCoV/Hong Kong/VM20001988/2020 | Hong Kong | 2020/1/30 | Human | Nasopharyngeal aspirate & Throat swab |
| EPI_ISL_412030 | BetaCoV/Hong Kong/VB20026565/2020 | Hong Kong | 2020/2/1 | Human | Nasopharyngeal aspirate & Throat swab |
| EPI_ISL_410545 | BetaCoV/Italy/INMI1-isl/2020 | Italy | 2020/1/29 | Human | Sputum |
| EPI_ISL_410546 | BetaCoV/Italy/INMI1-cs/2020 | Italy | 2020/1/31 | Human | Naso and pharyngeal swab |
| EPI_ISL_407084 | BetaCoV/Japan/AI/I-004/2020 | Japan | 2020/1/25 | Human | Pharyngeal swab |
| EPI_ISL_408665 | BetaCoV/Japan/TY-WK-012/2020 | Japan | 2020/1/29 | Human | Pharyngeal swab |
| EPI_ISL_408666 | BetaCoV/Japan/TY-WK-501/2020 | Japan | 2020/1/31 | Human | Pharyngeal swab |
| EPI_ISL_408667 | BetaCoV/Japan/TY-WK-521/2020 | Japan | 2020/1/31 | Human | Pharyngeal swab |
| EPI_ISL_408669 | BetaCoV/Japan/KY-V-029/2020 | Japan | 2020/1/29 | Human | Pharyngeal swab |
| EPI_ISL_410531 | BetaCoV/Japan/NA-20-05-1/2020 | Japan | 2020/1/25 | Human | Sputum |
| EPI_ISL_410532 | BetaCoV/Japan/OS-20-07-1/2020 | Japan | 2020/1/23 | Human | Sputum |
| EPI_ISL_410301 | BetaCoV/Nepal/61/2020 | Nepal | 2020/1/13 | Human | Oro-pharyngeal swab |
| EPI_ISL_406973 | BetaCoV/Singapore/1/2020 | Singapore | 2020/1/23 | Human | Sputum |
| EPI_ISL_407987 | BetaCoV/Singapore/2/2020 | Singapore | 2020/1/25 | Human |  |
| EPI_ISL_407988 | BetaCoV/Singapore/3/2020 | Singapore | 2020/2/1 | Human |  |
| EPI_ISL_410535 | BetaCoV/Singapore/4/2020 | Singapore | 2020/2/3 | Human |  |
| EPI_ISL_410536 | BetaCoV/Singapore/5/2020 | Singapore | 2020/2/6 | Human |  |
| EPI_ISL_410537 | BetaCoV/Singapore/6/2020 | Singapore | 2020/2/9 | Human |  |
| EPI_ISL_410713 | BetaCoV/Singapore/7/2020 | Singapore | 2020/1/27 | Human | Nasopharyngeal swab |
| EPI_ISL_410714 | BetaCoV/Singapore/8/2020 | Singapore | 2020/2/3 | Human | Nasopharyngeal swab |
| EPI_ISL_410715 | BetaCoV/Singapore/9/2020 | Singapore | 2020/2/4 | Human | Nasopharyngeal swab |
| EPI_ISL_410716 | BetaCoV/Singapore/10/2020 | Singapore | 2020/2/4 | Human | Nasopharyngeal swab |
| EPI_ISL_410719 | BetaCoV/Singapore/11/2020 | Singapore | 2020/2/2 | Human | Nasopharyngeal swab |
| EPI_ISL_407193 | BetaCoV/South Korea/KCDC03/2020 | South Korea | 2020/1/25 | Human | Throat swab |
| EPI_ISL_411929 | BetaCoV/South Korea/SNU01/2020 | South Korea | 2020/01 | Human |  |
| EPI_ISL_411951 | BetaCoV/Sweden/01/2020 | Sweden | 2020/2/7 | Human |  |
| EPI_ISL_406031 | BetaCoV/Taiwan/2/2020 | Taiwan | 2020/1/23 | Human |  |
| EPI_ISL_408489 | BetaCoV/Taiwan/NTU01/2020 | Taiwan | 2020/1/31 | Human | Sputum |
| EPI_ISL_410218 | BetaCov/Taiwan/NTU02/2020 | Taiwan | 2020/2/5 | Human | Sputum |
| EPI_ISL_411915 | BetaCoV/Taiwan/CGMH-CGU-01/2020 | Taiwan | 2020/1/25 | Human | Sputum |
| EPI_ISL_411926 | BetaCoV/Taiwan/3/2020 | Taiwan | 2020/1/24 | Human |  |
| EPI_ISL_411927 | BetaCoV/Taiwan/4/2020 | Taiwan | 2020/1/28 | Human |  |
| EPI_ISL_403962 | BetaCoV/Nonthaburi/61/2020 | Thailand | 2020/1/8 | Human | Nasopharyngeal swab and Throat swab |
| EPI_ISL_403963 | BetaCoV/Nonthaburi/74/2020 | Thailand | 2020/1/13 | Human | Nasopharyngeal swab and Throat swab |
| EPI_ISL_404253 | BetaCoV/USA/IL1/2020 | USA | 2020/1/21 | Human | sputum |
| EPI_ISL_404895 | BetaCoV/USA/WA1/2020 | USA | 2020/1/19 | Human | oropharyngeal swab |
| EPI_ISL_406034 | BetaCoV/USA/CA1/2020 | USA | 2020/1/23 | Human | nasopharyngeal swab |
| EPI_ISL_406036 | BetaCoV/USA/CA2/2020 | USA | 2020/1/22 | Human | nasopharyngeal swab |
| EPI_ISL_406223 | BetaCoV/USA/AZ1/2020 | USA | 2020/1/22 | Human | buccal swab |
| EPI_ISL_407214 | BetaCoV/USA/WA1-A12/2020 | USA | 2020/1/25 | Human | nasopharyngeal swab |
| EPI_ISL_407215 | BetaCoV/USA/WA1-F6/2020 | USA | 2020/1/25 | Human | Oropharyngeal swab |
| EPI_ISL_408008 | BetaCoV/USA/CA3/2020 | USA | 2020/1/29 | Human | nasopharyngeal swab |
| EPI_ISL_408009 | BetaCoV/USA/CA4/2020 | USA | 2020/1/29 | Human | oropharyngeal swab |
| EPI_ISL_408010 | BetaCoV/USA/CA5/2020 | USA | 2020/1/29 | Human | oropharyngeal swab |
| EPI_ISL_408670 | BetaCoV/USA/WI1/2020 | USA | 2020/1/31 | Human | nasopharyngeal swab |
| EPI_ISL_409067 | BetaCoV/USA/MA1/2020 | USA | 2020/1/29 | Human | oropharyngeal swab |
| EPI_ISL_410044 | BetaCoV/USA/CA6/2020 | USA | 2020/1/27 | Human | Respiratory swab |
| EPI_ISL_410045 | BetaCoV/USA/IL2/2020 | USA | 2020/1/28 | Human | Sputum |
| EPI_ISL_411954 | BetaCoV/USA/CA7/2020 | USA | 2020/2/6 | Human | Nasopharyngeal swab |
| EPI_ISL_411955 | BetaCoV/USA/CA8/2020 | USA | 2020/2/10 | Human | Nasopharyngeal swab |
| EPI_ISL_411956 | BetaCoV/USA/TX1/2020 | USA | 2020/2/11 | Human | Sputum |
| EPI_ISL_408668 | BetaCoV/Vietnam/VR03-38142/2020 | Vietnam | 2020/1/24 | Human |  |

**Table S7.** Detailed information of reference coronaviruses strains in this study.

| **Genus** | **Subgenus** | **Accession Number** | **Strain Name** | **Country** | **Date** | **Host** |
| --- | --- | --- | --- | --- | --- | --- |
| Alpha-Cov |  | NC002645 | Human coronavirus 229E |  |  | Homo sapiens |
| Alpha-Cov |  | NC005831 | Human coronavirus NL63 |  |  | Homo sapiens |
| Alpha-Cov |  | NC009657 | Scotophilus bat coronavirus 512 |  | 2005 | Bat |
| Alpha-Cov |  | NC010438 | Miniopterus bat coronavirus HKU8 | China-Hong Kong | 2008 | Miniopterus bat |
| Alpha-Cov |  | NC018871 | Rousettus bat coronavirus HKU10 | China | 2005 | Bat |
| Alpha-Cov |  | MK841495 | PEDV YZ | China | 2016 | Porcine |
| Alpha-Cov |  | MK841494 | PEDV SH | China | 2016 | Porcine |
| Alpha-Cov |  | KX499468 | TGEV AHHF | China | 2015 | Porcine |
| Beta-Cov | Embevovirus | AY391777 | Human coronavirus OC43 | UK |  | Homo sapiens |
| Beta-Cov | Embevovirus | KM349744 | Betacoronavirus HKU24-R05010I | China | 2012 | Rattus norvegicus |
| Beta-Cov | Embevovirus | FJ647223 | Murine coronavirus MHV-1 | USA |  | Mus musculus |
| Beta-Cov | Embevovirus | MK167038 | Human coronavirus HKU1 | USA | 2017 | Homo sapiens |
| Beta-Cov | Hibecovirus | KF636752 | Bat Hp-betacoronavirus/Zhejiang2013 | China-Zhejiang | 2013 | Hipposideros pratti |
| Beta-Cov | Merbecovirus | EF065505 | Bat coronavirus HKU4-1 | China-Guangdong |  | Bat |
| Beta-Cov | Merbecovirus | EF065509 | Bat coronavirus HKU5-1 | China-Guangdong |  | Bat |
| Beta-Cov | Merbecovirus | JX869059 | Human betacoronavirus 2c EMC/2012 | Saudi Arabia | 2012 | Homo sapiens |
| Beta-Cov | Merbecovirus | KC545386 | Betacoronavirus Erinaceus/VMC/DEU/2012 | Germany | 2012 | Erinaceus europaeus |
| Beta-Cov | Nobecovirus | KU762338 | Rousettus bat coronavirus GCCDC1 356 | China | 2014 | Rousettus leschenaulti |
| Beta-Cov | Nobecovirus | EF065513 | Bat coronavirus HKU9-1 | China-Guangdong |  | Bat |
| Beta-Cov | Nobecovirus | MK211379 | Coronavirus BtRt-BetaCoV/GX2018 | China | 2016 | Rhinolophus affinis |
| Beta-Cov | Sarbecovirus | AY508724 | SARS coronavirus NS-1 | China | 2003 | Homo sapiens |
| Beta-Cov | Sarbecovirus | AY485277 | SARS coronavirus Sino1-11 | China | 2003 | Homo sapiens |
| Beta-Cov | Sarbecovirus | AY390556 | SARS coronavirus GZ02 | China-Guangzhou | 2003 | Homo sapiens |
| Beta-Cov | Sarbecovirus | AY278489 | SARS coronavirus GD01 | China-Guangdong | 2003 | Homo sapiens |
| Beta-Cov | Sarbecovirus | KT444582 | SARS-like coronavirus WIV16 | China | 2013 | Rhinolophus sinicus |
| Beta-Cov | Sarbecovirus | KY417146 | Bat SARS-like coronavirus Rs4231 | China | 2013 | Rhinolophus sinicus |
| Beta-Cov | Sarbecovirus | KY417151 | Bat SARS-like coronavirus Rs7327 | China | 2014 | Rhinolophus sinicus |
| Beta-Cov | Sarbecovirus | KY417152 | Bat SARS-like coronavirus Rs9401 | China | 2015 | Rhinolophus sinicus |
| Beta-Cov | Sarbecovirus | MK211376 | Coronavirus BtRs-BetaCoV/YN2018B | China | 2016 | Rhinolophus sinicus |
| Beta-Cov | Sarbecovirus | KY770859 | Bat coronavirus Anlong-112 | China | 2013 | Rhinolophus sinicus |
| Beta-Cov | Sarbecovirus | KJ473816 | BtRs-BetaCoV/YN2013 | China | 2013 | Rhinolophus sinicus |
| Beta-Cov | Sarbecovirus | KY417145 | Bat SARS-like coronavirus Rf4092 | China | 2012 | Rhinilophus ferrumequinum |
| Beta-Cov | Sarbecovirus | MK211377 | Coronavirus BtRs-BetaCoV/YN2018C | China | 2016 | Rhinolophus affinis |
| Beta-Cov | Sarbecovirus | KY417142 | Bat SARS-like coronavirus As6526 | China | 2014 | Aselliscus stoliczkanus |
| Beta-Cov | Sarbecovirus | KY417148 | Bat SARS-like coronavirus Rs4247 | China | 2013 | Rhinolophus sinicus |
| Beta-Cov | Sarbecovirus | KJ473815 | BtRs-BetaCoV/GX2013 | China | 2013 | Rhinolophus sinicus |
| Beta-Cov | Sarbecovirus | JX993988 | Bat coronavirus Cp/Yunnan2011 | China | 2011 | Chaerephon plicata |
| Beta-Cov | Sarbecovirus | MK211374 | Coronavirus BtRl-BetaCoV/SC2018 | China | 2016 | Rhinolophus sp. |
| Beta-Cov | Sarbecovirus | GQ153547 | Bat SARS coronavirus HKU3-12 | China-Hong Kong | 2005 | Bat |
| Beta-Cov | Sarbecovirus | GQ153542 | Bat SARS coronavirus HKU3-7 | China-Hong Kong | 2007 | Bat |
| Beta-Cov | Sarbecovirus | MG772933 | Bat SARS-like coronavirus bat-SL-CoVZC45 | China-Zhejiang | 2017 | Rhinolophus sinicus |
| Beta-Cov | Sarbecovirus | MG772934 | Bat SARS-like coronavirus bat-SL-CoVZXC21 | China-Zhejiang | 2015 | Rhinolophus sinicus |

**Table S8.** Percentage of nucleotide sequence and amino acid sequence identity of SARS-CoV-2 and reference CoV strains.

| **Genomic Region** | **Strain** | **SARS-CoV-2 WHU01** | **Bat-CoV RaTG13** | **Bat-SL-CoV ZXC21** | **Bat-SL-CoV ZC45** | **SARS-CoV GD01** | **MERS-CoV** |
| --- | --- | --- | --- | --- | --- | --- | --- |
| **Full-length genome** | SARS-CoV-2 WHU01 |  | **96.2** | **88.0** | **88.1** | **79.7** | **54.3** |
|  | Bat-CoV RaTG13 |  |  | **88.0** | **88.0** | **79.5** | **54.3** |
|  | Bat-SL-CoV ZXC21 |  |  |  | **97.5** | **81.1** | **54.1** |
|  | Bat-SL-CoV ZC45 |  |  |  |  | **81.0** | **54.1** |
|  | SARS-CoV GD01 |  |  |  |  |  | **53.1** |
|  | MERS-CoV |  |  |  |  |  |  |
|  | | | | | | | |
| **S gene** | SARS-CoV-2 WHU01 |  | **93.1** | **76.9** | **77.5** | **74.2** | **48.5** |
|  | Bat-CoV RaTG13 | **97.7** |  | **77.7** | **77.9** | **74.1** | **48.2** |
|  | Bat-SL-CoV ZXC21 | **81.6** | **81.3** |  | **97.0** | **73.8** | **48.7** |
|  | Bat-SL-CoV ZC45 | **82.2** | **81.8** | **98.7** |  | **73.8** | **48.8** |
|  | SARS-CoV GD01 | **77.3** | **77.6** | **77.3** | **77.5** |  | **47.9** |
|  | MERS-CoV | **28.8** | **28.8** | **29.2** | **29.1** | **28.9** |  |
|  | | | | | | | |
| **E gene** | SARS-CoV-2 WHU01 |  | **98.7** | **98.7** | **98.7** | **94.3** | **52.4** |
|  | Bat-CoV RaTG13 | **100.0** |  | **100.0** | **100.0** | **93.9** | **53.3** |
|  | Bat-SL-CoV ZXC21 | **100.0** | **100.0** |  | **100.0** | **93.9** | **53.3** |
|  | Bat-SL-CoV ZC45 | **100.0** | **100.0** | **100.0** |  | **93.9** | **53.3** |
|  | SARS-CoV GD01 | **94.7** | **94.7** | **94.7** | **94.7** |  | **49.1** |
|  | MERS-CoV | **41.3** | **41.3** | **41.3** | **41.3** | **40.8** |  |
|  | | | | | | | |
| **M gene** | SARS-CoV-2 WHU01 |  | **95.9** | **93.4** | **93.4** | **85.4** | **51.2** |
|  | Bat-CoV RaTG13 | **98.6** |  | **93.1** | **93.1** | **83.8** | **50.8** |
|  | Bat-SL-CoV ZXC21 | **98.6** | **99.1** |  | **100.0** | **83.9** | **51.1** |
|  | Bat-SL-CoV ZC45 | **98.6** | **99.1** | **100.0** |  | **83.9** | **51.1** |
|  | SARS-CoV GD01 | **89.6** | **91.0** | **90.0** | **90.0** |  | **50.0** |
|  | MERS-CoV | **39.7** | **40.2** | **39.7** | **39.7** | **42.0** |  |
|  | | | | | | | |
| **N gene** | SARS-CoV-2 WHU01 |  | **96.9** | **91.2** | **91.1** | **88.8** | **54.9** |
|  | Bat-CoV RaTG13 | **99.0** |  | **91.2** | **91.4** | **88.5** | **54.9** |
|  | Bat-SL-CoV ZXC21 | **94.3** | **94.3** |  | **98.7** | **90.9** | **55.7** |
|  | Bat-SL-CoV ZC45 | **94.3** | **94.3** | **99.3** |  | **91.0** | **55.6** |
|  | SARS-CoV GD01 | **91.2** | **91.2** | **92.1** | **92.4** |  | **54.5** |
|  | MERS-CoV | **48.0** | **48.0** | **47.0** | **47.0** | **47.8** |  |

The upper right indicates the nucleotide sequence identity. The lower left indicates the amino acid sequence identity.

**Table S9.** Sequence variability among structural proteins of SARS-CoV-2 clinical isolates.

| **Protein** | **Mutation** | **Strain Name** |
| --- | --- | --- |
| **Spike protein (S1/NTD)** | F32I | BetaCoV/Wuhan/HBCDC-HB-01/2019 \| EPI_ISL_402132 |
|  | H49Y | BetaCoV/Guangdong/20SF028/2020 \| EPI_ISL_403936 |
|  |  | BetaCoV/Guangdong/20SF040/2020 \| EPI_ISL_403937 |
|  |  | BetaCoV/Jiangsu/JS02/2020 \| EPI_ISL_411952 |
|  |  | BetaCoV/USA/CA5/2020 \| EPI_ISL_408010 |
|  | S221W | BetaCoV/South Korea/SNU01/2020 \| EPI_ISL_411929 |
|  | S247R | BetaCoV/Australia/VIC01/2020 \| EPI_ISL_406844 |
| **Spike protein (S1/RBD)** | Deletion of F^342^ | BetaCoV/England/01/2020 \| EPI_ISL_407071 |
|  | N354D | BetaCoV/Shenzhen/SZTH-004/2020 \| EPI_ISL_406595 |
|  | D364Y | BetaCoV/Shenzhen/SZTH-004/2020 \| EPI_ISL_406595 |
|  | V367F | BetaCoV/France/IDF0372/2020 \| EPI_ISL_406596 |
|  |  | BetaCoV/France/IDF0373/2020 \| EPI_ISL_406597 |
|  |  | BetaCoV/France/IDF0372-isl/2020 \| EPI_ISL_410720 |
|  |  | BetaCoV/France/IDF0386-islP1/2020 \| EPI_ISL_411219 |
|  |  | BetaCoV/France/IDF0386-islP3/2020 \| EPI_ISL_411220 |
| **Spike protein (S1)** | D614G | BetaCoV/Germany/BavPat1/2020 \| EPI_ISL_406862 |
|  |  | BetaCoV/Hong Kong/VM20001061/2020 \| EPI_ISL_412028 |
| **Spike protein (S2)** | F797C | BetaCoV/Sweden/01/2020 \| EPI_ISL_411951 |
|  | Deletion of N^824^ | BetaCoV/USA/IL1/2020 \| EPI_ISL_404253 |
|  | V860Q | BetaCoV/Beijing/IVDC-BJ-005/2020 \| EPI_ISL_408485 |
|  | L861K | BetaCoV/Beijing/IVDC-BJ-005/2020 \| EPI_ISL_408485 |
|  | F970S | BetaCoV/Beijing/IVDC-BJ-005/2020 \| EPI_ISL_408485 |
|  | Deletion of ^1081^ICHD^1084^ | BetaCoV/Shanghai/IVDC-SH-001/2020 \| EPI_ISL_408483 |
|  | G1093A | BetaCoV/Shanghai/IVDC-SH-001/2020 \| EPI_ISL_408483 |
|  | V1129L | BetaCoV/Shenzhen/SZTH-001/2020 \| EPI_ISL_406592 |
|  | P1143L | BetaCoV/Australia/QLD02/2020 \| EPI_ISL_407896 |
|  | V1177A | BetaCoV/Shanghai/IVDC-SH-001/2020 \| EPI_ISL_408483 |
|  | E1262G | BetaCoV/Shenzhen/SZTH-001/2020 \| EPI_ISL_406592 |
| **Envelope protein** | L37H | BetaCoV/South Korea/SNU01/2020 \| EPI_ISL_411929 |
| **Matrix protein** | Deletion of A^69^ | BetaCoV/USA/IL1/2020 \| EPI_ISL_404253 |
|  | G78A | BetaCoV/Shenzhen/SZTH-001/2020 \| EPI_ISL_406592 |
|  | D209H | BetaCoV/Singapore/2/2020 \| EPI_ISL_407987 |
|  |  | BetaCoV/Singapore/6/2020 \| EPI_ISL_410537 |
| **Nucleocapsid protein** | T148I | BetaCoV/Shenzhen/SZTH-004/2020 \| EPI_ISL_406595 |
|  | S194L | BetaCoV/Foshan/20SF207/2020 \| EPI_ISL_406534 |
|  |  | BetaCoV/Shenzhen/SZTH-003/2020 \| EPI_ISL_406594 |
|  |  | BetaCoV/USA/CA3/2020 \| EPI_ISL_408008 |
|  |  | BetaCoV/USA/CA4/2020 \| EPI_ISL_408009 |
|  |  | BetaCoV/USA/MA1/2020 \| EPI_ISL_409067 |
|  | Deletion of S^194^ | BetaCoV/USA/IL1/2020 \| EPI_ISL_404253 |
|  | S202N | BetaCoV/Australia/QLD01/2020 \| EPI_ISL_407894 |
|  |  | BetaCoV/Australia/QLD02/2020 \| EPI_ISL_407896 |
|  |  | BetaCoV/Australia/QLD03/2020 \| EPI_ISL_410717 |
|  |  | BetaCoV/Australia/QLD04/2020 \| EPI_ISL_410718 |
|  |  | BetaCoV/Singapore/4/2020 \| EPI_ISL_410535 |
|  |  | BetaCoV/USA/CA7/2020 \| EPI_ISL_411954 |
|  | P344S | BetaCoV/Guangzhou/20SF206/2020 \| EPI_ISL_406533 |
|  |  | BetaCoV/Japan/KY-V-029/2020 \| EPI_ISL_408669 |
