# Supplementary material for: Immunoinformatic Analysis of T- and B-Cell Epitopes for SARS-CoV-2 Vaccine Design
Source: Vaccines (Basel). 2020 Jul 3;8(3):355. doi: 10.3390/vaccines8030355 (PMC7563688; doi:10.3390/vaccines8030355)
Supplement: Supplementary file 1 [file vaccines-08-00355-s001.zip › vaccines-833738-supplementary-for proof.docx]

1. Supplementary Methods

1.1. Sequence Datasets

In all, 138 genomes of SARS-CoV-2 isolated from 16 countries were downloaded from GISAID (<http://gisaid.org/>), and the detailed information is summarized in Table S6. The additional 42 reference coronavirus sequences used in this study were collected from the NCBI (Table S7).

1.2. Phylogenetic Tree and Selection Pressure Analysis

Sequences were aligned by ClustalW implemented in MEGA 7 software. A maximum-likelihood (ML) tree was reconstructed with *s*, *e*, *m*, and *n* nucleotide sequences, respectively, using MEGA 7 with the GTR model and 1000 bootstrap replicates. The *p*-distance method was used to reconstruct a neighbor-joining (NJ) tree with 1000 bootstrap replicates. The selection pressure analysis of genomes was determined by calculating the difference between non-synonymous substitution (dN) and synonymous substitution (dS) rates for the aligned genes by using MEGA7 software, respectively. The calculated value of dN-dS was used to evaluate the selection pressure (dN-dS > 0: positive selection; dN-dS < 0: purifying selection; dN-dS = 0: neutral selection) [1].

1.3. Structural Analysis

The secondary structure of the SARS-CoV-2 S protein was predicted by PSIPRED [2]. 3D structure homology modeling was reconstructed based on the SARS-CoV-2 S protein structure (PDB ID: 6VYB) in SWISS-MODEL and was displayed with Pymol 1.8.0.3 ([www.pymol.org](http://www.pymol.org)).

2. Supplementary Results

2.1. Genetic and Phylogenetic Tree Analysis

Pairwise‐alignments of the SARS-CoV-2 genome (reference strain: SARS-CoV-2 WHU01) with other coronavirus genomes among the same family showed that SARS-CoV-2 WHU01 shared 96.2% nucleotide identity with bat-CoV RaTG13, 88% with bat-SL-CoV ZC45 or ZXC21, and 79.7% with SARS-CoV GD01, but only 54.3% identity with MERS-CoV (Table S8).

For the protein sequence alignments, the M, N, and E proteins derived from SARS-CoV-2 WHU01 all shared more than 89% amino acid (aa) identity with the same proteins derived from SARS-CoV GD01 or the three bat-CoVs (bat-CoV RaTG13, bat-SL-CoV ZC45, and bat-SL-CoV ZXC21). In particular, the E protein shared 94.7% and 100% aa identities with the E proteins derived from SARS-CoV GD01 and the three bat-CoVs, respectively (Table S8). Interestingly, the SARS-CoV-2 WHU01 S protein sequence shared 77.3% aa identity with the SARS-CoV GD01 S protein, but 97.7% identity with the S protein of bat-CoV RaTG13. Only 29 residues were variable between the SARS-CoV-2 S protein and the bat-CoV RaTG13 S protein, and 21 out of the 29 residues were present in the RBD domain of the S1 subunit (Figure S5A). This result indicated that the RBD domain is one of the hotspots in the virus evolution. Notably, previous studies have revealed that two critical residues (Gln493 and Asn501, see Figure S5A) in the receptor-binding motif (RBM) of the SARS-CoV-2 S protein provided an even more favorable interaction with human ACE2 [3].

Next, to trace the origin of SARS-CoV-2, we employed the phylogenetic method of neighbor joining (NJ) and the *s*, *e*, *m*, or *n* as reference genes for constructing phylogenetic trees. The results demonstrated that the cluster of SARS-CoV-2 is genetically related to the bat-SL-CoV (Figure S2). Similar results were obtained (Figure S3) with another phylogenetic method (ML, see Supplementary Methods), These results indicated that the bat-SL-CoV is a potential origin in the evolution of SARS-CoV-2.

2.2. Genetic Variation of SARS-CoV-2

Comparison of the genome sequences of 138 SARS-CoV-2 isolates revealed that all the SARS-CoV-2 strains share 99% nucleotide identity and the four structural proteins (S, E, M, and N) of this virus were highly conserved, particularly the E protein (Table S9). These results suggested that SARS-CoV-2 has not yet acquired substantial mutations within this subset. S protein sequences were identical in 116 isolates but had either single or multiple point mutations or deletions occurring at 23 positions in the remaining 22 isolates. Four of the 23 positions were present in the RBD; however, none of the mutations occurred in the RBM, indicating that the RBM is strictly conserved among these 138 SARS-CoV-2 isolates (Table S9). Amino acid mutations among the four structural proteins of SARS-CoV-2 isolates are summarized in Table S9. A special strain, South Korea/SNU01/2020, isolated from a patient living in South Korea needs to be highlighted since this strain harbored two mutations that did not present in the rest of the SARS-CoV-2 strains. One of the two mutations occurred in the E protein (L37H) and this was also the only mutation that has so far been discovered in E proteins, and the other mutation occurred in the S protein (S221W). The two mutations probably were critical for the transmission and pathogenesis of SARS-CoV-2 [4]. Furthermore, the selection pressure of SARS-CoV-2 was analyzed by calculating the difference dN and dS rates for the *s, e, m*, and *n* genes, respectively. The overall mean differences of dN-dS were 0.166 for the *s* gene, 0 for the *e* gene, −0.056 for the *m* gene, and −0.104 for the *n* gene. The result suggested that the *s* gene was under positive selection and the *e* gene was under neutral selection, whereas the *m* and *n* genes were under purifying selection.

References

1. Hughes, A.L.; Nei, M. Nucleotide substitution at major histocompatibility complex class II loci: evidence for overdominant selection. *Proc. Natl Acad Sci* **1989**, *86*, 958-62.
2. Daniel, W.A.B.; Federico, M.; Tim, C.O.N; Kevin, B.; David, T.J. Scalable web services for the PSIPRED Protein Analysis Workbench. Nucleic acids research **2013**; *41*, W349–W357.
3. Wan, Y.; Shang, J.; Graham, R.; Baric, R.S.; Li, F. Receptor recognition by novel coronavirus from Wuhan: An analysis based on decade-long structural studies of SARS. *J. Virol.* **2020**, doi: 10.1128/JVI.00127-20.
4. Tang, X.; Wu, C.; Li, X., Song, Y.; Yao, X.; Wu, X.; Duan, Y.; Zhang, H.; Wang, Y.; Qian, Z.; et al. On the origin and continuing evolution of SARS-CoV-2. *Natl. Sci. Rev.* **2020**, 7, 1012–1023.
